# The Role of Anti-angiogenesis in the Treatment Landscape of Non-small Cell Lung Cancer – New Combinational Approaches and Strategies of Neovessel Inhibition

**DOI:** 10.3389/fcell.2020.610903

**Published:** 2021-01-05

**Authors:** Sophia Daum, Hannes Hagen, Erin Naismith, Dominik Wolf, Andreas Pircher

**Affiliations:** ^1^Internal Medicine V, Department of Hematology and Oncology, Medical University Innsbruck, Innsbruck, Austria; ^2^Medical Clinic 3, Department of Oncology, Hematology, Immunoncology and Rheumatology, University Hospital Bonn (UKB), Bonn, Germany

**Keywords:** non-small cell lung cancer, angiogenesis, vascular endothelial growth factor, tumor microenvironment, tumor endothelial cells, immunotherapy, combinational therapy

## Abstract

Tumor progression depends primarily on vascular supply, which is facilitated by angiogenic activity within the malignant tissue. Non-small cell lung cancer (NSCLC) is a highly vascularized tumor, and inhibition of angiogenesis was projected to be a promising therapeutic approach. Over a decade ago, the first anti-angiogenic agents were approved for advanced stage NSCLC patients, however, they only produced a marginal clinical benefit. Explanations why anti-angiogenic therapies only show modest effects include the highly adaptive tumor microenvironment (TME) as well as the less understood characteristics of the tumor vasculature. Today, advanced methods of in-depth characterization of the NSCLC TME by single cell RNA sequencing (scRNA-Seq) and preclinical observations enable a detailed characterization of individual cancer landscapes, allowing new aspects for a more individualized inhibition of angiogenesis to be identified. Furthermore, the tumor vasculature itself is composed of several cellular subtypes, which closely interact with other cellular components of the TME, and show distinct biological functions such as immune regulation, proliferation, and organization of the extracellular matrix. With these new insights, combinational approaches including chemotherapy, anti- angiogenic and immunotherapy can be developed to yield a more target-oriented anti-tumor treatment in NSCLC. Recently, anti-angiogenic agents were also shown to induce the formation of high endothelial venules (HEVs), which are essential for the formation of tertiary lymphoid structures, and key components in triggering anti-tumor immunity. In this review, we will summarize the current knowledge of tumor-angiogenesis and corresponding anti-angiogenic therapies, as well as new aspects concerning characterization of tumor-associated vessels and the resulting new strategies for anti-angiogenic therapies and vessel inhibition in NSCLC. We will further discuss why anti-angiogenic therapies form an interesting backbone strategy for combinational therapies and how anti-angiogenic approaches could be further developed in a more personalized tumor-oriented fashion with focus on NSCLC.

## Introduction

Angiogenesis is regulated by the balance of pro-angiogenic and anti-angiogenic factors present in a tissue, if vascular remodeling is required this balance shifts to an activating state, called the “angiogenic switch” (Bergers and Benjamin, 2003). In progressing tumors, a similar activated angiogenic phenotype occurs which promotes endothelial cell (EC) proliferation, migration, elongation and dissemination of metastases to distant organs (Teleanu et al., 2019). These findings proposed inhibiting angiogenesis as a highly potent anti-cancer therapeutical approach and have intensified the research for agents to hamper vessel formation in diverse tumors over the past decades (Augustine et al., 2019). Conclusively, inhibiting pro-angiogenic molecules including vascular endothelial growth factors (VEGFs) or their cognate receptors (VEGFRs), served as anti-angiogenic therapy approaches in advanced stage NSCLC patients, as well as other cancer entities, at the beginning of this century (Sandler et al., 2006) and reviewed in Jayson et al. (2016). High expectations for these anti-cancer drugs were shattered rapidly as they exhibited only marginal benefits in early clinical trials due to the acquisition of evasive or primary resistance mechanisms consequently leading to a transient therapy benefit. Therapy failure could be attributed to the interplay of adaptive mechanisms of the TME (e.g., eliciting compensatory angiogenic pathways) and its interacting cellular compartments including TECs. In the previous years, a more detailed characterization identified the (tumor) endothelium as a heterogeneous cell population with distinct functional and organ-specific phenotypes indicating multiple pathological features of the tumor vasculature (Rafii et al., 2016). In addition to tumor endothelial heterogeneity, other vessel formation processes alongside vascular sprouting, such as vessel co-option or vasculogenic mimicry (VM) were less acknowledged in NSCLC but may play an important role in anti-angiogenic therapy resistance. Furthermore, the ability of tumors to compensate for absent signaling molecules by activating alternative pathways represents another resistance mechanism. The inhibition of VEGF/VEGFR, for example, was shown to prompt tumors to sustain angiogenesis via the secretion of substitute factors such as PDGF (Crawford et al., 2009), bFGF (Babina and Turner, 2017) and angiopoietin-2 (Rigamonti et al., 2014), or by the recruitment of pro-angiogenic cells such as tryptase secreting mast cells (Wroblewski et al., 2017) thus, resisting single-target therapies. Previous studies using dual or multi-target antibodies which simultaneously inhibit several angiogenic signals exhibited an incremental anti-angiogenic efficacy in different tumor types (Li et al., 2016; Peterson et al., 2016; Liu et al., 2018; Hosaka et al., 2020). However, many processes and factors contributing to inefficacy and resistance to angiogenesis inhibitors, in particular those involving the tumor endothelium, remain ambiguous.

The transient combinational efficacy of anti-angiogenic agents and chemotherapy (in first-line, as well as in second-line therapy) could possibly be attributed to a “vascular normalization” phenotype. Nevertheless, the time window of vessel re-organization and normalization is not well understood in the clinical setting but could play a major role in the transmission of chemical agents directly to the tumor, thereby enhancing anti-cancer efficacy (Johnson et al., 2004; Sandler et al., 2006; Garon et al., 2014; Reck et al., 2014). Additionally, cancer immunotherapies which inhibit immune checkpoints (ICs) such as programmed cell death protein 1/programmed cell death 1 ligand 1 (PD1/PD-L1) and cytotoxic T-lymphocyte-associated protein 4 (CTLA4) have become landmarks in cancer treatment. The interaction of tumor vasculature with immune cells has a severe impact on the responsiveness and immunodeficiency of the tumor. Vascular normalization due to VEGF-inhibiting therapy exhibited increased lymphocyte infiltration and T-cell activation which, combined with immune checkpoint inhibitors (ICI), elicited an improved anti-tumor immunity in preclinical trials (Allen et al., 2017; Schmittnaegel et al., 2017). Additionally, combinational therapy of anti-angiogenic agents and ICI resulted in the formation of HEVs, which enhances activation of circulating B- and T-cells by mediating migration into secondary lymphoid organs (Ager and May, 2015). When surrounded by dense B- and T-cell rich areas, HEV can further adapt to tertiary lymphoid structures (TLS) thereby triggering potent anti-tumor immunity, which can significantly improve patient outcomes (Martinet and Girard, 2013). We are confronted with a network of considerable aspects when it comes to anti-angiogenic therapy, many of which still require thorough investigation. Further characterization of the TME and the associated endothelium can help improve anti-angiogenic therapies and optimize the proposed powerful synergic efficacy of combinational therapeutical approaches in NSCLC.

## The Role of Tumor Angiogenesis in NSCLC

Physiological angiogenesis has already been characterized in detail and previously reviewed elsewhere (Góth et al., 2003; Senger and Davis, 2011). The process of tumor angiogenesis, which occurs early during tumor progression, is similar to physiological vessel formation, but with differences in regulation and grade of activity (Hanahan and Folkman, 1996; Raica et al., 2009; Hanahan and Weinberg, 2011). Firstly, tumor associated ECs acquire a chronic activated state, the “angiogenic switch,” a result of the upregulation of angiogenic receptors and activation of the PI3K-AKT signaling axis (Phung et al., 2006). This activation results in increased proliferation, survival and migration, leading to distortion of the basement membrane as well as pericyte coverage in the tumor vasculature (Hida et al., 2016). Consequently, TECs exhibit dysregulated behavior and polarization resulting in leaky, hemorrhagic, and dysfunctional vessels. Thus, oxygen levels, nutrient availability and waste disposal is diminished, which has severe effects on the TME (Colegio et al., 2014; Sanctis et al., 2018). Furthermore, dysfunctional TECs severely impact lymphocyte adhesion, trafficking and migration to the local tissue, resulting in a highly immunosuppressive TME (Fridman et al., 2012).

Additionally, the tumor stroma, which consists of a mix of resident fibroblasts and pericytes as well as bone-marrow derived tumor infiltrating leukocytes (e.g., macrophages and mast cells), regulates angiogenesis. M2 polarized tumor associated macrophages can either directly activate angiogenesis by releasing VEGF, bFGF and PlGF or indirectly via the release of matrix-metalloproteinases (MMPs), which in turn remodel the extracellular matrix for an enhanced endothelial migration (Kessenbrock et al., 2010; Olson and Joyce, 2015). Fibroblasts, as well as myeloid derived suppressor cells (MDSCs) promote angiogenesis through expression of growth factors such as VEGF and bFGF (Shi et al., 2017). CSF-1, a cytokine crucial for the survival and differentiation of monocytes and macrophages, mediates the recruitment of MDSCs into the tumor niche, which in turn increases angiogenesis due to growth factor release (Shojaei et al., 2007). By blocking the CSF-1 signaling in combination with anti-VEGFR2 therapy, tumor growth could be markedly decreased in murine lung carcinoma models (Priceman et al., 2010). Mast cells comprise a major compartment of inflammatory cells present in the TME and exhibit important regulatory features regarding angiogenesis (Ribatti and Crivellato, 2012). Their granules contain various proteases, cytokines and growth factors including pro-angiogenic molecules such as VEGF, bFGF, PDGF and the potent angiogenic factor tryptase, which is released upon activation of IgE or c-kit receptors (Ribatti and Ranieri, 2015). Tryptase induces vascularization and vessel tube formation by stimulating proliferation of ECs and activation of MMPs (Ribatti and Crivellato, 2012). In NSCLC the number of tryptase positive MCs linearly correlates with microvascular density, confirming the important role of this enzyme in regulating tumor angiogenesis (Ibaraki et al., 2005; Carlini et al., 2010). Inhibition of c-kit and its ligand SCF could hamper mast cell infiltration into the TME, preventing degranulation and thereby producing a synergizing anti-angiogenic effect (Huang et al., 2008; Overed-Sayer et al., 2020).

Current vessel-inhibiting therapies for treating advanced NSCLC mainly focus on repressing the process of vessel sprouting predominantly triggered by VEGF signaling. In the past years, however, non-angiogenic processes in the TME have gained attention as they are suggested to significantly contribute to tumor progression while being resistant to traditional angiogenesis inhibitors. In highly vascularized organs such as the lung, it was observed that cancer cells start to grow along existing vessels to preserve access to essential nutrients and gases without the need to form new vasculature. This process is referred to as vessel co-option (Pezzella et al., 1997; Coelho et al., 2017). In contrast to the chaotic growth of angiogenic tumor vessels, co-opted vasculature remains well organized as deduced from normal tissues (Adighibe et al., 2006). So far, vessel co-option is suggested to result, at least in part, of differential mitochondrial metabolism, but it may also involve reduced inflammation (Donnem et al., 2013). The ECs of co-opted vessels experience severe molecular changes during this process, for e.g., starting to express angiopoietin-2, which results in strong regression of vessels in the tumor core (Coelho et al., 2017). Thereupon, the tumor core becomes hypoxic, which consequently activates the angiogenic switch in tumor vessels (Holash et al., 1999). *In vitro* studies of glioma cells suggest that tumor cells that facilitate vessel co-option are dependent on the endoplasmic reticulum based stress sensing protein IRE1 (Auf et al., 2010). Furthermore the MMP-activating protein B2R was shown to serve as a chemoattractant during the migration of glioma cells towards blood vessels (Montana and Sontheimer, 2011). Finally, CDC42, a protein involved in actin-dependent formation of cytoplasmatic extensions, together with CD44, a protein crucial for establishing cell-cell contact, enable the connection between tumor cells and vessel covering pericytes for vessel co-option (Caspani et al., 2014).

Another non-angiogenic mechanism termed “VM” describes the process where cancer cells gain endothelial abilities to form their own circulatory network consisting of microvascular tubes to preserve blood supply (Pinto et al., 2016). So far, the molecular mechanism behind VM is not yet understood, however, it appears that VE-cadherin, the most prominent receptor on ECs, may play an important role. VE-cadherin on tumor cells can activate PI3K through the ERK1/ERK2 pathway which subsequently activates the metalloproteases MMP14 and pro-MMP2, resulting in remodeling of the ECM to enable cancer cells to be reorganized into vessel-like tubes (Paulis et al., 2010; Delgado-Bellido et al., 2017). VM networks resemble embryonic vasculogenesis, referring to a highly aggressive tumor cell phenotype that converted to an embryonic-like, undifferentiated state to facilitate tube formation (Maniotis et al., 1999). Gene expression analysis of VM networks in aggressive melanoma identified genes correlated with various cellular phenotypes such as fibroblasts, ECs and epithelial cells (Bittner et al., 2000; Seftor et al., 2002a,b). Tumors positive for VM show an increased expression of the ECM component laminin5γ2 and several MMPs, underlining the importance of ECM remodeling for initiating and promoting this non-angiogenic process (Seftor et al., 2001). Furthermore, VM is associated with poor prognosis as it is mainly observed in aggressive forms of melanoma and lung metastases (Williamson et al., 2016). Taking the potent impact of these non-angiogenic processes in cancer progression into consideration, may help us explain the occurring resistance of lung tumors to VEGF-inhibitors (Döme et al., 2007; Bergers and Hanahan, 2008).

In summary, the pathological features of tumor-associated ECs and non-ECs which result in a complex cancer promoting TME are diverse, and consequently contribute to therapy failure of angiogenesis inhibitors as well as other therapy approaches in a remarkable fashion. To better understand the biological mechanisms behind drug resistance or lack of clinical benefit, further investigation into the detailed characterization of the endothelial compartment in the TME are essential.

## Traditional Methods for Vessel Inhibition in NSCLC

Currently used anti-angiogenic agents have been developed and approved for clinical application after intense study of their molecular, cellular, and physiological mode of action using various experimental approaches. In the following part we summarize currently available methods for investigating tumor angiogenesis as well as anti-angiogenic agents that have already been accepted for treating NSCLC.

### Methods to Study (Tumor) Angiogenesis

Experimental models remain the cornerstone for investigating tumor angiogenesis and the development of new anti-angiogenic therapies. As vessel sprouting is a multistep process there is a wide array of assays which enable individual evaluation of different stages, and each possesses specific advantages and disadvantages (Shahid et al., 2017; Stryker et al., 2019). To unravel these complex processes, it is crucial to understand the analytical potential of each model. *In vitro* methods represent the fundamental evaluation of tumor angiogenesis including basic functional analysis such as proliferation, migration, and tube formation. The big advantages of *in vitro* assays are their simplicity, high reproducibility, and cost effectiveness, while the disadvantages include the incomplete representation of the cellular heterogeneity and prevailing conditions present in human organs. Although findings from *in vitro* assays may never be conclusive alone, they serve as a preliminary projection of angiogenic processes upon treatment of choice and provide first insights into a testing hypothesis.

*Ex vivo* assays such as the thoracic aorta ring and retina angiogenesis methods represent the link between *in vitro* and *in vivo* analysis. Here, functional vasculature fragments of aorta/retina derived from mice or rats are immersed in a three-dimensional culturing system for evaluating vessel sprouting outgrowth under specific conditions. The advantage of this method over *in vitro* assays is the preservation of original EC properties within the tissue that are normally modified due to isolation processes and repeated passaging. The absence of blood flow and circulating EC progenitors or other factors constitute the main disadvantages of these methods.

For more accurate information regarding angiogenic processes upon treatment in a biological system or to perform long-term studies, *in vivo* methods are necessary. The most common systems to investigate angiogenesis in a living organism are the chicken chorioallantoic membrane (CAM) assay, matrigel plugs, and tumor xenograft models. CAM assays, which have already been in use for decades, utilize chorioallantoic membranes of fertilized chicken eggs to evaluate angiogenic processes. While this method is cost effective, highly reproducible and the outcomes are easily visualized, it must be taken into consideration that vessel growth is evaluated during developmental stages, which can affect studies investigating mechanisms in mature vasculature. Matrigel plug assays enable the use of an *in vitro* tool in an *in vivo* setting. Here, vascular growth is evaluated by injection of matrigel, a synthesized substrate resembling basement membrane matrix, into an animal model which allows easy stimulation, subsequent excision, and investigation of the plug with, for example, immunohistological stainings. Compared with CAM assays, the matrigel plug can be used in more analytical methods and provides a fast and reliable representation of angiogenic processes in a biological system. Nevertheless, this method may require more replicates due to higher variability of results and is therefore more expensive. Lastly, transplantation xenografts represent the most advanced method to investigate tumor angiogenesis in a living organism. Tumor cells, mostly of human origin, are injected into immunodeficient mice to induce formation of a cancer mass that can be further treated and monitored for changes regarding tumor angiogenesis. This method most suitably reflects the pathological mechanism of vessel growth *in vivo* in the presence of blood circulation, as well as diverse environmental factors. Furthermore, it enables the long-term study of diverse processes associated with angiogenesis that are observed in a biological system such as tissue invasion, distant metastasis formation as well as non-angiogenic processes like vessel co-option and VM, which are known to promote resistance mechanisms in various cancers. Aside from the ethical aspect, a considerable disadvantage of this method is the incomplete or lacking representation of the immune system due to immunosuppression of the study organism.

Examining which experimental assay is most suitable for investigating a chosen angiogenic process under certain conditions, necessitates extensive deliberation with the desired endpoint, required technical equipment, level of experimental throughput, cost, and ethics kept in mind. Additionally, the complexity of angiogenesis cannot be unraveled using a single analytical method but the thought-out application of multiple overlapping analyses, ranging from cellular to physiological levels, are necessary to obtain robust findings worth testing in the clinical setting.

### Anti-angiogenic Therapies Approved for Treating NSCLC

In 2004, the first VEGFA-inhibiting antibody, bevacizumab, was approved for use in advanced colorectal cancer in combination with chemotherapy and was followed in 2006 in NSCLC (Sandler et al., 2006). Since then, diverse anti-angiogenic antibodies or tyrosine kinase inhibitors (TKIs) have been developed, which block either VEGF-A binding to the receptor or directly inhibit VEGFR-2 to hamper vascularization in tumors. VEGF-pathway inhibition has a broad anti-angiogenic effect in tumors: (1) it primarily inhibits vessel growth which induces regional cancer cell death and delays progression of the tumor rather than diminishing its size (Escudier et al., 2007); (2) it induces EC apoptosis as VEGF acts as a survival factor on the endothelium by activating BCL2, Akt signaling, or apoptosis inhibitors (Gerber et al., 1998; Fujio and Walsh, 1999); (3) it blocks the recruitment of hematopoietic or endothelial progenitor cells for new vessel formation which provides an essential function in neovascularization in growing tumors (Rafii et al., 2002; Bertolini et al., 2006). Angiogenesis inhibitors in combination with either chemotherapeutics, targeted therapies or ICI, in first or second-line therapies in NSCLC, have exhibited improved efficacy and feasible safety, which significantly improved response rates and prolonged progression free survival (PFS) in a large number of patients. Currently three anti-angiogenic agents, namely bevacizumab, ramucirumab and nintedanib are FDA/EMA approved for use in advanced stage NSCLC and are summarized in Table 1 (Hall et al., 2015; Alshangiti et al., 2018; Janning and Loges, 2018) while more are in clinical testing. Despite the remarkable clinical benefits of these combinational approaches on response rate and PFS, the overall survival (OS) benefits were modest due to acquired drug resistance. It is important to mention that in most lung cancer studies anti-angiogenic therapy is administered until the onset of severe drug related adverse effects or disease progression. So far, there is only preclinical evidence that discontinued angiogenesis inhibition results in TME reorganization and perhaps causes a rebound effect of tumor angiogenesis. In tumor and healthy mouse models, it could be shown that anti-VEGF therapy withdrawal resulted in rapid tissue revascularization and long lasting structural changes including vessel hyper-permeability and increased metastasis in the diseased cohort (Yang et al., 2016). The treatment-triggered hypoxia which induces angiogenesis especially during therapy-withdrawal is one possible explanation to this tumor promoting off-drug effect. The benefit of continuous anti-angiogenic therapy beyond disease progression in the clinical setting was first analyzed in a phase 3b trail in 2018 which included 485 advanced NSCLC patients (Gridelli et al., 2018). Here, bevacizumab was administered in addition to standard of care therapy beyond disease progression. While, the treatment continuation of bevacizumab yielded no substantial therapy benefit, improvements in efficacy, and no new safety signals were observed. Based on these findings, the approach of continuous angiogenesis inhibition should be further investigated but may be recommended at a certain degree in the future. Nevertheless, treatment decisions should be based on individual therapeutic efficacy, which needs to be tracked throughout the entire therapy. However, the absence of reliable biomarkers with predictive features for anti-angiogenic therapies hamper further therapy improvement, thus molecular screening for markers associated with tumor angiogenesis is currently of great value.

**TABLE 1:** Clinical studies of anti-angiogenesis based therapies in NSCLC that led to FDA/EMA approval.

Line	Population	Therapy-combination	Experimental arms	Phase	References
First	Stage IIIB-IV NSCLC	AAT + ChT	Bevacizumab + Carboplatin + Paclitaxel (*n* = 434)	III	Sandler et al., 2006
			Carboplatin + Paclitaxel (*n* = 444)		
	Advanced non-squamous NSCLC	AAT + ChT	Bevacizumab (low) + Cisplatin + Gemcitabine (*n* = 345)	III	Reck et al., 2009; Reck et al., 2010
			Bevacizumab (high) + Cisplatin + Gemcitabine (*n* = 351)		
			Cisplatin + Gemcitabine (*n* = 347)		
	Stage IIIB-IV non-squamous NSCLC	AAT + ChT	Bevacizumab + standard chemotherapy (*n* = 2212)	IV	Crinò et al., 2010
	Locally advanced, recurrent or metastatic NSCLC	AAT + ChT	Bevacizumab + Platin-based chemotherapy (*n* = 1313)	II/III	Soria et al., 2013
			Platin-based chemotherapy (*n* = 881)		
	Stage IIIB-IV EGFR-mutated non-squamous NSCLC	AAT + TT	Bevacizumab + Erlotinib (*n* = 75)	II	Seto et al., 2014; Yamamoto et al., 2018
			Erlotinib (*n* = 77)		
	Stage IIIB-IV EGFR-mutated non-squamous NSCLC	AAT + TT	Bevacizumab + Erlotinib (*n* = 114)	III	Saito et al., 2019
			Erlotinib (*n* = 114)		
	Stage IV EGFR-mutated NSCLC	AAT + TT	Ramucirumab + Erlotinib (*n* = 224)	II	Nakagawa et al., 2019
			Erlotinib (*n* = 225)		
	Metastatic non-squamous NSCLC	AAT + ChT + ICI	Bevacizumab + Atezolizumab + Carboplatin + Paclitaxel (*n* = 356)	III	Socinski et al., 2018
			Bevacizumab + Carboplatin + Paclitaxel (*n* = 336)		
Second	Stage IV squamous and non-squamous NSCLC	AAT + ChT	Ramucirumab + Docetaxel (*n* = 1253)	III	Garon et al., 2014; Reck et al., 2017
			Docetaxel (*n* = 625)		
	Stage IIIB-IV NSCLC	AAT + ChT	Nintedanib + Docetaxel (*n* = 655)	III	Reck et al., 2014; Novello et al., 2015
			Docetaxel (*n* = 654)		

## New Molecular Candidates to Predict and Track Anti-Angiogenic Efficacy in NSCLC

As previously mentioned, there is a great need for biomarkers to predict and track anti-angiogenic therapy efficacy, to help overcome innate and acquired resistance as it is still the main obstacle that restrains clinical success (Bergers and Hanahan, 2008). So far, predictive angiogenesis-associated biomarkers in NSCLC are lacking, highlighting the need for further investigation to improve this anti-tumor approach.

In a recent study, it was demonstrated that immunohistochemically confirmed TTF-1 expression in advanced non-squamous NSCLC samples, which is a known prognostic biomarker of lung adenocarcinomas, could be linked to therapy success of bevacizumab in combination with pemetrexed plus platinum derivatives (Takeuchi et al., 2018). TTF-1 positive tumors exhibited enhanced clinical benefits when bevacizumab was combined with the basic therapy whereas TTF-1 negative tumors did not benefit from this addition.

Furthermore, despite the previous results of the IMpower150 study, where significant clinical benefits of bevacizumab in combination with ICI and chemotherapy were shown, regardless of PDL-L1 expression, a phase 1b study by Herbst et al. observed contrary results. They reported a beneficial efficacy of ramucirumab in combination with the PD-L1 inhibitor pembrolizumab especially in patients with a PD-L1 expression above 50% (Herbst et al., 2019). According to this, PD-L1 expression remains a predictive marker of ICI therapy or ICI therapy in combination with anti-angiogenesis agents in NSCLC. Qiu et al. recently examined the benefit of anti-angiogenic therapies (bevacizumab, anlotinib or others) with anti-PD-L1 agents (nivolumab or pembrolizumab) in a real-world study including 69 NSCLC patients. Subgroup analyses in the cohort revealed that the response and PFS of this combinational therapy was significantly higher when it was administered as first-line therapy compared to other lines of treatment, and when the therapy was initiated within the first 6 months of diagnosis compared to later time points (Qiu et al., 2020). Additionally, patients with EGFR wildtype tumors exhibited significantly prolonged PFS after the combinational therapy compared to patients with EGFR mutated tumors. Interestingly, no correlation between PDL-1 expression levels and the efficacy of this combinational therapy has been observed so far, however, follow up will be continued. In short, these study results can help to optimize the use of anti-angiogenic agents in combination with PD-L1 inhibitors, however, more factors need to be investigated to yield an optimal benefit.

Another potent multi-targeted anti-angiogenic TKI, anlotinib, has already shown profound benefits as third-line combinational therapy in advanced NSCLC (Han et al., 2018a,b). A transcriptomics study of an anlotinib-resistant lung cancer cell line, indicated that CXCL2, a cytokine involved in wound healing and angiogenesis, was also involved in anlotinib-resistance (Lu et al., 2019a). *In vitro* assays demonstrated that exogenous CXCL2 could recover anti-angiogenic-induced inhibition of migration and invasion and prevent apoptosis of anlotinib-resistant cells. Furthermore, in a retrospective analysis, anlotinib-induced decrease of the inflammatory cytokine CCL2 in serum correlated with prolonged PFS and OS (Lu et al., 2019b). Nevertheless, resistance and poor response to anlotinib hinder drug efficacy. While the underlying mechanisms are still unknown, elevated serum-levels of two angiogenesis-related markers KLK5 and L1CAM were recently correlated with poor response to anlotinib (Lu et al., 2019b).

Easily available predictive biomarkers, e.g., liquid biopsy, which allow the continuous track of response to angiogenesis inhibition are highly desired to optimize efficacy, as most of the current methods involve invasive procedures (biopsy or surgery) which limit analytical accessibility.

Several studies suggested a potential prognostic value of VEGF in NSCLC but so far investigations into circulating VEGF levels have not yielded consistent results (Rodríguez Garzotto et al., 2016). In the E4599 study, high VEGF levels in pretreatment plasma of 878 patients with advanced stage NSCLC, who received combinational treatment of bevacizumab plus chemotherapy, correlated with increased overall response but had no predictive outcome on survival (Dowlati et al., 2008). Another study observed contrary results when baseline plasma biomarkers of 303 non-squamous NSCLC patients undergoing similar therapy were evaluated (Mok et al., 2014). Here, baseline VEGFA levels in the plasma correlated with prolonged PFS and OS but showed no association with response rates to the therapy. The predictive value of VEGF or other proangiogenic factors on anti-angiogenic drug response is a highly discussed matter revealing vastly variable results. This is partly due to analytical variability, including sample collection and handling, as well as the disagreements regarding the most suitable sample choice for evaluating circulating factors (Rodríguez Garzotto et al., 2016). For example, serum or platelet rich plasma may not adequately represent the physiological VEGF level as it has been shown that the clotting processes initiates VEGF release in platelets (Webb et al., 1998). Moreover, the pathological situation can impact VEGF levels, as patients with more advanced tumors or several metastatic tumor sites exhibit a higher baseline level of plasma VEGFA, suggesting that VEGFA is linked to the tumor burden (Mok et al., 2014). Previously proposed correlations of circulating angiogenic factor levels with anti-angiogenic therapy efficacy in lung cancer seem to reflect tumor biology thus, have an important prognostic role rather than to be predictive (Crohns et al., 2010). The observed trend of increasing circulating factors in response to angiogenesis inhibition on one hand was shown to depend considerably on the TME and may represent therapy-induced hypoxia (Zaman et al., 2006; Kut et al., 2007). On the other hand, high VEGFA levels could also be attributed to TP53 mutated lung tumors which correlated with improved efficacy of bevacizumab (Schwaederlé et al., 2015). A currently identified alternative biomarker for bevacizumab-based chemotherapy combinations in patients with advanced NSCLC is CXCL16. In the analyzed sera of 40 advanced staged NSCLC patients therapy-induced decrease of CXCL16 levels correlated with prolonged OS compared with patients exhibiting only moderate decrement (Shibata et al., 2020).

However, confirming if any of these molecular markers indeed exhibit adequate predictive features necessitates further investigation. New aspects of processes which promote tumor angiogenesis, and a better understanding of the endothelium as driving force can help identify reliable biomarkers and overcome therapy failure in NSCLC.

## Mechanisms of Tumor Vascularization in NSCLC

There are several mechanisms on both the cellular and environmental levels which can promote vessel formation in human tumors, many of which are not yet been completely elucidated. Although angiogenesis may represent the most important part of tumor vascularization, other processes that result in perfusion of the tumor tissue should be investigated in more detail and considered when designing new anti-angiogenic approaches in NSCLC.

**FIGURE 1:**
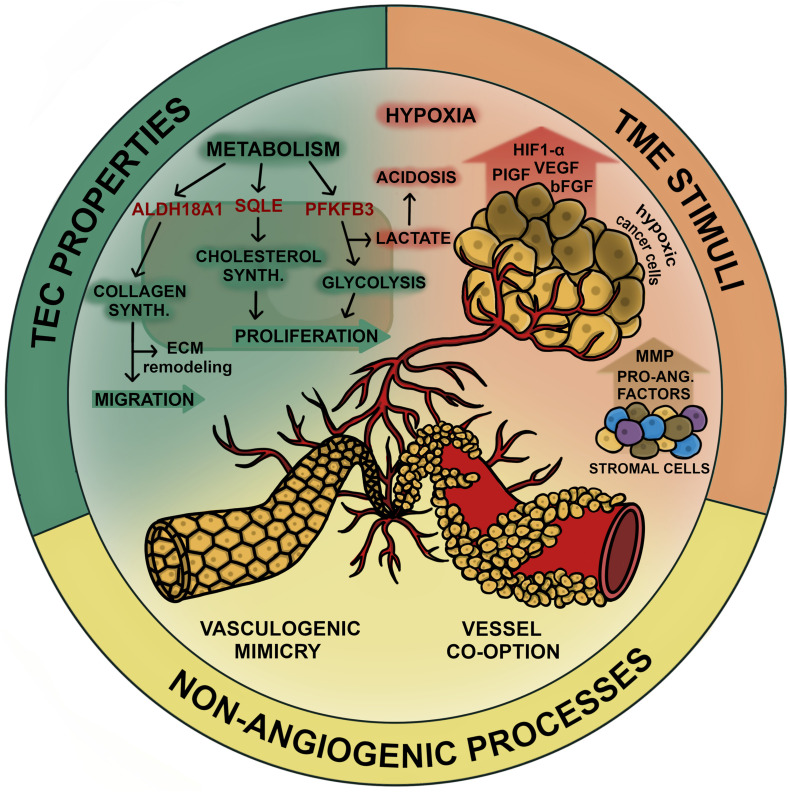
Mechanisms of tumor vascularization in NSCLC. Tumor vascularization in lung cancer can be promoted by various processes which overlap during cancer progression. In general tumor vascularization/angiogenesis can be stimulated on the cellular level (TEC properties), the environmental level (TME stimuli) or facilitated in absence of angiogenic signaling (non-angiogenic processes). TECs exhibit upregulated metabolism to enable high angiogenic activity which includes processes involved in proliferation (cholesterol synthesis and glycolysis) and processes that enable migration via ECM remodeling (collagen synthesis). Potential targets involved in these pathways (SQLE, PFKFB3, and ALDH18A1, respectively) are considered to increase the angiogenic potential of TECs in NSCLC. Hypoxia and acidosis induced by high levels of lactate due to upregulated glycolysis constitute to a highly pro-angiogenic tumor environment. Angiogenesis stimulating factors (VEGF, bFGF, PDGF, HIF-1α, tryptase, and MMPs) are released by both, cancer cells and stromal cells, including fibroblasts, pericytes, tumor associated macrophages and ECs. Non-angiogenic processes constitute to tumor vascularization and are inaccessible for anti-angiogenic agents, thus contributing to therapy resistance. VM comprises the formation of tubular structures arising from cancer cells that gain endothelial like properties to maintain vascular supply during cancer progression. Another mechanism of cancer cells to persist in circulation is to grow along existing vasculature, which is referred to as vessel co-option. In this figure we summarized the various mechanism of tumor vascularization that should be considered when targeting the inhibition of tumor vessels in NSCLC.

In the following part we summarize various levels of tumor vascularization that may represent new targets for vessel inhibition in NSCLC. All mentioned mechanisms are summarized in Figure 1.

### TEC Characteristics That Promote Tumor Vascularization in NSCLC

The endothelium is postulated to be a large contributor to the therapeutic efficacy of anti-angiogenic therapies, and therefore represents a possible source of therapy response or failure. It is well known that the process of angiogenesis is comprised of different EC phenotypes which execute distinct functions. During the elongation of the sprouting vessel VEGF-sensitive tip ECs migrate into avascular tissue regions, thus leading the proliferating trailing stalk ECs, which built up the growing vessel. Newly formed vasculature finally adapts a mature and quiescent phenotype referred to as phalanx ECs (Carmeliet and Jain, 2011; Betz et al., 2016). The EC phenotypes involved are highly dynamic and can reprogram the gene expression to meet their current physiological requirements. However, the tumor endothelium was not studied in depth and a recent single-cell RNA sequencing (scRNA-Seq) study identified even more EC phenotypes from both healthy and tumor tissue from lung cancer samples as already known, indicating a much more complex phenotypic heterogeneity of the (tumor) vasculature than initially presumed (Goveia et al., 2020). Interestingly, although phenotype proportions differed strongly between analyzed NSCLC patients, they collectively observed a low abundance of tip and proliferating TECs, which represent the main targets of traditional anti-angiogenic therapy. Furthermore, they identified a so-far-unknown tumor exclusive phenotype of activated postcapillary vein EC that upregulated features known from HEVs in inflamed tissues such as immunomodulatory factors and ribosomal proteins. The unexpected finding that activated and proliferating TECs only represent a minority of the pathological EC phenotypes found in NSCLC, allows us to reconsider currently used anti-angiogenic therapy as less of a vessel-inhibiting strategy, and more of a strategy to modulate the higher proportion of mature TECs into potent participants of tumor surveillance.

**FIGURE 2:**
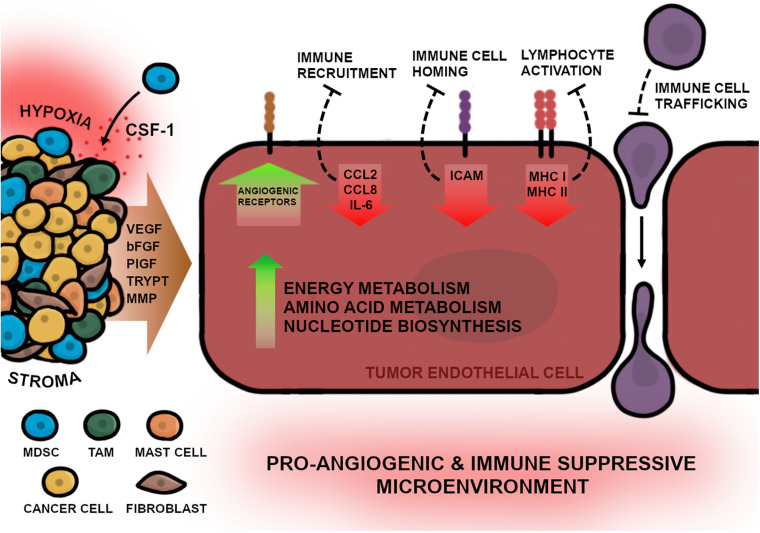
The multifaced picture of TECs in NSCLC. TECs possess features that enable continuous angiogenic activity for progressing vascularization of the tumor. These features are ensured by genetical changes in the tumor endothelium that are triggered by diverse stimuli of the TME e.g., hypoxia and growth factor release. The stroma, consisting of various cells, promote angiogenesis by directly releasing signaling molecules into the adjacent tissue, thereby stimulating TECs. Fibroblasts and myeloid derived suppressor cells (MDSCs) activate angiogenesis by releasing VEGF and bFGF into the TME. Additionally, CSF-1 molecules, expressed by cancer cells, further recruit MDSCs into the tumor niche. Tumor associated macrophages (TAMs) can directly induce angiogenesis by releasing VEGF, bFGF, and PlGF, or indirectly by releasing matrix metalloproteinases (MMPs) which promote endothelial migration. Mast cells secrete tryptase (TRYPT) into the TME which stimulates EC proliferation and enables ECM remodeling. Furthermore, to facilitate enhanced angiogenesis, TECs upregulate the surface expression of angiogenic receptors as well as increase metabolic activity including energy and amino acid metabolism and the biosynthesis of nucleotides. In addition to the high angiogenic activity, TECs can directly suppress inflammatory responses by downregulation of inflammatory cytokines for immune cell recruitment (CCL2, CCL8, and IL-6), receptors required for immune cell homing (ICAM) or lymphocyte activation (MHC I and MHC II) which results in impaired immune cell trafficking and migration into the TME. In summary the complex interaction of tumor-protecting environmental conditions and the pathological features of TECs lead to a pro-angiogenic and immune suppressive TME in NSCLC.

In order to develop new angiogenesis-inhibiting therapies, the molecular differences between physiological and pathological ECs will need to be elaborated. Genetically TEC and NEC phenotypes significantly differ in gene expression affecting diverse cellular mechanisms such as proliferation, migration, inflammation, and angiogenesis (Figure 2). Previous studies have shown that one key feature of TECs is a highly active metabolism, which permits pathological processes as increased proliferation and angiogenesis (Cantelmo et al., 2016). TECs exhibit upregulated glycolysis due to elevated expression of 6-phosphofructo-2-kinase/fructose-2,6-biphosphatase 3 (PFKFB3), which regulates proliferation and migration during tumor angiogenesis (van der Veldt et al., 2012; de Bock et al., 2013). Hyperglycolytic TECs subsequently release high amounts of lactate into the environment, which in turn, further stimulates EC proliferation and angiogenesis (Annan et al., 2019). It could be demonstrated that inhibition of PFKFB3 resulted in improved drug efficacy and decreased metastatic events in tumor mouse models (Cantelmo et al., 2016). Another study in xenograft NSCLC mouse models exhibited that PFKFB3 mRNA silencing in combination with docetaxel results in a chemoenhancing effect and increases anti-cancer efficacy compared with monotherapies alone (Chowdhury et al., 2017). Furthermore, to sustain upregulated proliferative capacity, TECs exhibit elevated nucleotide biosynthesis including upstream pathways that are involved in serine and lipid synthesis (Cantelmo et al., 2016; Bruning et al., 2018; Li et al., 2019). In addition, Lambrechts et al. (2018) showed that MYC-targets, which are involved in transcription processes, were most upregulated in TECs of human NSCLC samples. Interestingly, c-MYC expression induces angiogenesis in combination with HIF-1α and VEGF (Lee and Wu, 2015) and recruits tryptase positive mast cells into the tumor niche (Soucek et al., 2007), therefore, MYC inhibition may have a potential anti-cancer effect.

Focusing on endothelial metabolism in cancer, a recent study could identify at least two metabolic signatures which are highly upregulated in angiogenic endothelium and TECs. One for proliferation, which includes gene sets associated with biomass production e.g., glycolysis, TCA cycle, fatty acid oxidation, cholesterol biosynthesis and amino acid metabolism, and one for remodeling of the extracellular matrix including gene sets for collagen biosynthesis in particular proline synthesis (Rohlenova et al., 2020). These results educed two new possible metabolic targets to hamper tumor angiogenesis; aldehyde dehydrogenase 18 family member A1 (ALDH18A1), an enzyme essential for *de novo* biosynthesis of proline; and squalene epoxidase (SQLE), the rate-limiting enzyme in cholesterol biosynthesis. Silencing of ALDH18A as well as SQLE impaired EC proliferation, migration and vessel sprouting in *in vitro* assays. Summarized, targeting endothelial metabolism in cancer is an interesting therapeutic option that could possibly assist an anti-angiogenic approach for treating NSCLC.

Another key feature of TECs in lung cancer is the downregulation of inflammatory responses thus contributing to tumor-associated immune escape. Single-cell analysis of NSCLC samples identified the most downregulated genes of the tumor endothelium in connection to inflammation, which included CCL2, CCL18, and IL6, essential for immune cell recruitment; MHC I and II, essential for immune cell activation; and ICAM, required for immune cell homing (Lambrechts et al., 2018). As the endothelium represents the primary connection between the immune system and tumor cells, these results indicate the important role of TECs in immunomodulatory processes that hamper anti-tumor immunity. It has been demonstrated that angiogenesis inhibition can restore proinflammatory surface proteins on TECs during a therapy-induced process termed “vessel normalization” (Goveia et al., 2020). Vessel normalization not only improves immune cell activation and infiltration, but is also suggested to enhance drug delivery to the tumor sites, thus improving its efficacy (Allen et al., 2017; Schmittnaegel et al., 2017). Additionally, combinational therapy of angiogenesis inhibitors and immunotherapy (anti-PD-L1) in previous studies could elicit the formation of unique blood vessels in treated tumors that resemble HEVs typically found in lymphoid tissues, which implicated increased treatment efficacy (Allen et al., 2017; Schmittnaegel et al., 2017). HEVs can mediate immune cell adhesion and migration into the tumor, which may be important for bypassing TEC-induced immune escape (Ager and May, 2015). In the already discussed scRNA-Seq study by Goveia et al., they demonstrated that VEGFR inhibition could induce vessel normalization by shifting invasive, low immunogenic TEC phenotypes to a more quiescent, immune-modulatory phenotype resembling HEVs (Goveia et al., 2020). These remarkable observations indicate that TECs comprise the ability to transform into HEVs to promote immune cell infiltration into the tumor and induce a potent anti-tumor response. This extends the previous observations of favorable synergistic effects of immune therapy in combination with angiogenesis inhibitors in NSCLC, especially when it results in HEV formation. Furthermore, direct induction of HEV formation could be a promising new strategy in anti-angiogenic approaches that may attain great clinical importance. However, currently there are no reliable biomarkers to track the process of vessel normalization or HEV formation in NSCLC which could help to predict and optimize this new treatment strategy.

### Non-angiogenic Mechanisms in Association With Neovessel Inhibition in NSCL

As mentioned above, in some cases tumor vascularization can be facilitated by non-ECs which adapt certain properties to sustain access to the circulation, which may support anti-angiogenic drug resistance. During tumor progression, processes that lead to vascularization of the malignant tissue can vary locally as well as temporarily and involve angiogenic as well as non-angiogenic mechanisms even in the same lesion (Bridgeman et al., 2017). In lung tumors, where non-angiogenic tumor growth occurs most commonly, previous studies primarily located non-angiogenic processes in the tumor periphery, whereas angiogenesis is typically localized in the hypoxic tumor core (Pezzella et al., 1997; Donnem et al., 2018). Here, we briefly discuss the impact of non-angiogenic processes in NSCLC on anti-angiogenic drug efficacy based on previous studies.

VEGF-A inhibition using bevacizumab failed to inhibit VM in breast cancer cells *in vitro*, furthermore, sunitinib, a multi targeting anti-VEGFR inhibitor, even promoted VM in breast cancer mouse models (Dey et al., 2015; Sun et al., 2017). Additionally it could be demonstrated that VM in NSCLC depends on expression of Sema4D and its receptor plexinB1 which activate RhoA and downstream ROCK, comprising an already known angiogenesis-promoting process in tumors (Basile et al., 2006). Inhibition of Sema4D or downregulation of plexinB1 resulted in RhoA/ROCK pathway inhibition and could reduce VM formation in human NSCLC cell lines (Xia et al., 2019). Although the role of VM in NSCLC is not fully understood, previous observations suggest that it may contribute to anti-angiogenic therapy failure and may serve as an option to treat aggressive lung tumors.

Vessel co-option on the other hand is a common phenomenon especially observed in lung metastases when tumor cells start to invade perivascular tissues (Jensen, 2016). Anti-angiogenic therapy with sunitinib could induce a switch from angiogenic vessel formation to vessel co-option in a lung metastatic mouse model, which ultimately resulted in sunitinib resistance (Bridgeman et al., 2017). Unfortunately, regulative mechanisms of vessel co-option in human tumors remain unknown in large part, however, predicting the occurrence of either VM or vessel co-option could be a useful tactic to prevent anti-angiogenic drug resistance in some patients. According to these and other results, it could be confirmed that non-angiogenic tumors contribute to anti-angiogenic therapy resistance which reveals the undoubted importance of targeting both angiogenic, but also non-angiogenic vessel growth to treat NSCLC (Donnem et al., 2018).

## New Aspects for Vessel Inhibition in NSCLC

Increasing knowledge of the physiological processes of tumor vascularization in addition to traditional angiogenesis has enlightened a variety of adaptive mechanisms which can promote anti-angiogenic therapy resistances. This awareness fortifies the necessity for alternative anti-angiogenic agents besides traditional anti-VEGF therapy.

### New Targets for Vessel Inhibition in NSCLC

As previously examined, tumor angiogenesis depends on upregulated metabolic activity e.g., elevated cholesterol levels in TECs. Cholesterol not only represents a fundamental structural component of cell membranes and serves as precursor for several steroid hormones, it is also crucial for membrane function and angiogenic signaling, making it a favorable target for tumor vessel inhibition (Lyu et al., 2017). Inhibition of intracellular cholesterol trafficking with anti-inflammatory drug chepharantine was shown to hamper angiogenesis and tumor growth in lung cancer xenograft mice while improving anti-tumor activity of standard chemotherapeutics (Lyu et al., 2017). Another study has shown that pharmacological lowering of intracellular cholesterol levels with pitavastatin could reduce growth and migration and induced apoptosis in human lung tumor-associated ECs *in vitro* (Hu et al., 2020). *In vivo* experiments using lung cancer xenograft mice exhibited that pitavastatin-treatment could completely arrest tumor growth in these animals when combined with cisplatin and delayed tumor growth and impaired angiogenesis in cisplatin-resistant mouse models.

Another potential angiogenic target for cancer treatment is tie1. While the second tie receptor, tie2, is well characterized as a regulator during late stages of angiogenesis (e.g. vascular maturation or in quiescent ECs) via the angiopoetin/tie signaling pathway, an associated ligand for the less studied tie1, has not yet been identified (Augustin et al., 2009). In contrast to tie2, tie1 is upregulated in angiogenic vessels and downregulated in quiescent ECs and is somehow involved in the regulation of the angiopoietin/tie signaling cascade (Sato et al., 1995; Kim et al., 2016; Korhonen et al., 2016; La Porta et al., 2018). As tie1 is also upregulated in intratumoral vasculature, its deletion on ECs successfully produced a potent anti-angiogenic effect in different cancers (Kaipainen et al., 1994; Aguayo et al., 2001). In fact, EC-specific deletion of tie1 in lung carcinoma and melanoma mouse models resulted in delayed cancer growth, predominantly in late-stage tumors (La Porta et al., 2018). Furthermore, it inhibited neovessel sprouting and a reduced intratumoral vessel density, while the remaining mature vasculature became strongly normalized, which limited further metastatic formation. These findings, and the fact that tie1 expression is increased in angiogenic endothelium compared with resting vasculature, presents tie1 as a highly potent angiogenic target, especially in the treatment of advanced staged NSCLC.

Another considerable strategy of anti-angiogenic therapy could include targeting micro RNAs (miRNAs) as they represent a new paradigm in molecular cancer therapy. The impact of miRNAs in post-transcriptional regulation has already been associated with pathways involved in cancer and vascular disease as summarized in Sun et al., 2018. The following studies evaluated the potential role of specific angiogenesis-related miRNAs as targets in lung cancer. Hsu et al. observed that miR-23a, a micro RNA known to be hypoxia-associated, was overexpressed in exosomes of oxygen depleted CL1-5 lung cancer cells (Hsu et al., 2017). Furthermore, these cancer-cell derived exosomes could induce angiogenesis via HIF-1α signaling *in vitro* when internalized by HUVECs. Additionally, miR-23a transfection increased permeability and transendothelial migration of cancer cells *in vitro* by downregulation of the tight junction protein ZO-1 and stimulated neovascularization and tumor growth *in vivo* in CL1-5 xenograft mice, proposing it to be an appealing target for anti-angiogenic therapy. Upregulation of miR-195 in squamous lung cancer cells *in vitro* on the other hand could be associated with impaired VEGF expression and hampered migration and invasion, thereby facilitating a tumor-suppressive function. Additionally, overexpression of miR-195 in HUVECs was observed to inhibit tube formation and reduced the expression of VEGF, which hampered their angiogenesis activity *in vitro* (Liu et al., 2019).

As it is an essential process during vessel growth, targeting ECM remodeling may also be an interesting approach to inhibit tumor angiogenesis in NSCLC. The most prominent enzymes involved in this process are matrix-metalloporoteinases (MMPs) which are inhibited under physiological conditions by tissue inhibitors of metalloproteinases (TIMPs). miR-130b could be identified as a promotor of MMP-2 activity and invasion of NSCLC cancer cells *in vitro* by downregulation of TIMP-2. Additionally, it could be observed that miR-130b was significantly upregulated in tumor tissue of NSCLC patients with vascular cancer cell invasion (Hirono et al., 2019). According to these findings, targeting miR-130b could be a strategy to impede angiogenesis and cancer cell invasion in lung cancer.

Uribesalgo et al. suggested targeting the apelin signaling pathway to inhibit tumor vessel formation in lung cancer (Uribesalgo et al., 2019). Apelin is a conserved peptide involved in developmental angiogenesis and is also upregulated in ECs within the TME. Previous studies could associate high apelin levels with a poor clinical outcome in patients with NSCLC (Györffy et al., 2010). In murine lung cancer models, apelin knockout reduced tumor burden and prolonged survival by inhibiting VEGF, TGF-β1, and TNF-α and simultaneously decreased MDSC infiltration in the TME (Uribesalgo et al., 2019). The combination of pharmacological inhibition of apelin with the anti-angiogenic drug sunitinib in lung cancer and mammary cancer mouse models, significantly delayed tumor growth and could almost double the survival, even in the KRAS driven or p53 mutated tumors, when compared with sunitinib treatment alone. Finally, apelin loss also reduced vessel density and prevented sunitinib-induced hypoxia and poor vessel structure in the TME. Conclusively, apelin inhibition may provide a potent synergistic anti-tumor effect when combined with anti-angiogenic agents, while, and most importantly, avoiding therapy-induced hypoxia of the TME, thus decreasing the chance of metastases, and bypassing potential therapy resistances.

### New Therapy Approaches for Vessel Inhibition in NSCLC

Single-target anti-angiogenic agents have already shown their limitations in clinical settings (Jayson et al., 2016). Even in combination with other therapy approaches like standard chemotherapy or immune therapy, treatment success remains largely marginal. Targeting several pro-angiogenic molecules with recombinant fusion proteins could therefore increase the anti-angiogenic effect of such therapies. Zhang et al. (2020) could successfully establish a multi-epitope peptibody containing bFGF and VEGF sequences, which could provoke a potent anti-bFGF/VEGF response by inhibiting proliferation and migration of lung cancer cells as well as HUVECs *in vitro*. When injected into lung cancer mouse models, autologous generated anti-peptibody antibodies inhibited tumor progression and angiogenesis and decreased expression of bFGF, VEGFA and PDGF in the tumor tissue. Targeting angiogenesis with fusion proteins exhibited potent anti-tumor efficacy in murine models and may represent a new approach for vessel inhibition in NSCLC, especially in combination with other therapy agents aimed at important angiogenic factors, previously discussed potential TEC specific markers or cellular mechanisms (Table 2).

**TABLE 2:** Pro-angiogenic factors involved in angiogenesis.

Factor	Abbreviation	Angiogenic function
Vascular endothelial growth factor	VEGF	Inducing angiogenesis by stimulating proliferation, survival, and migration of ECs
Basic fibroblast growth factor	bFGF	Inducing angiogenesis by stimulating proliferation and migration of ECs and extracellular matrix degradation
Hypoxia-inducible factor 1-alpha	HIF-1α	Regulating proangiogenic factor expression under oxygen depletion
Platelet-derived growth factor	PDGF	Inducing angiogenesis by stimulating proliferation, migration and tube formation of ECs and regulating VEGF signaling
Tryptase	TRYPT	Inducing angiogenesis by stimulating proliferation of ECs and vascular tube formation
Colony stimulating factor 1	CSF-1	Inducing release of proangiogenic factors by MDSC
Placental growth factor	PlGF	Inducing angiogenesis by stimulating proliferation, survival and migration of ECs and recruiting proangiogenic macrophages
Angiopoietin-2	Ang2	Regulating neovascular remodeling, vessel maturation and sensitizing ECs to cytokines
Matrix metalloproteases	MMPs	Remodeling of ECM for endothelial migration
6-Phosphofructo-2-Kinase/Fructose-2,6-Biphosphatase	PFKFB3	Metabolic regulation of proliferation and migration in ECs
MYC		Inducing angiogenesis in combination with HIF-1α and VEGF
Aldehyde dehydrogenase 18 family member A1	ALDH18A1	Regulating collagen biosynthesis for remodeling of ECM for endothelial migration
Squalene epoxidase	SQLE	Regulating cholesterol biosynthesis for EC proliferation
Apelin	APLN	Involved in developmental angiogenesis and expressed in angiogenic ECs
miR-23a		Inducing angiogenesis via HIF-1α signaling
Fibronectin/EIIIB		Remodeling of ECM for endothelial migration

The instability of tumor vessels due to morphological abnormalities (e.g., incomplete pericyte coverage) impedes drug delivery to the local lesion. Although anti-angiogenic therapy can temporarily restore tissue perfusion and drug delivery by vascular normalization, treatment withdrawal often results in vessel hyper-permeability and can even induce a rebound effect of tumor angiogenesis (Yang et al., 2016). As continuous inhibition of angiogenesis remains difficult to implement for health or economic reasons, an alternative or more independent delivery system of anti-angiogenic agents could help to overcome these issues. Nanomaterials have become an emerging field in cancer therapy in recent years, as their unique molecular properties make them suitable targeted drug delivery-systems. Physiochemically, these nanoparticles match the size of inter-endothelial junctions of blood vessels in the TME and therefore increase permeation and retention (EPR) resulting in a passive drug delivery (Chauhan and Jain, 2013). Nanomaterials such as liposomes or nanotube carbon structures are used to deliver anti-angiogenic agents and improve drug specificity while reducing cytotoxic side effects, drug clearance and resistance mechanisms in the treatment of NSCLC (Seshadri and Ramamurthi, 2018). In the past, studies using biodegradable polymers as nanocarriers to deliver chemotherapeutics and targeted drugs exhibited significant anti-tumor efficacy *in vitro* and *in vivo*. For example, paclitaxel encapsulated aldehyde polyethylene glycol-polylactide (PEG-PLGA) conjugated to a VEGFR2-inhibiting peptide showed increased internalization in HUVECs *in vitro* as well as potent activity against breast cancer models *in vivo* (Yu et al., 2010). Although there are several peptide motifs that are suggested to target tumor endothelium such as RGD or NGR which can bind integrin heterodimers CD51 and CD61, or aminopeptidase N, respectively, their targeting with nanomaterial is not yet applied for treating NSCLC (Sakurai et al., 2019). Furthermore, non-angiogenic mechanisms such as VM or vessel co-option could also represent possible targets for nanomaterial-based therapy as the EPR effect of such molecules could help to overcome delivery and infiltration issues of traditional cancer therapeutics. However, nanotherapeutics may provide a new potential anti-angiogenic therapeutical approach, but as already discussed, there is still a need for more specific biomarkers to exclusively target tumor vasculature in an organ specific manner. Taking this into consideration, chimeric antigen receptor (CAR) T-cell therapy, which serves as personalized immune therapy using autologous T-lymphocytes, engineered to target specific antigens present in a tumor, could be used to exclusively eliminate TECs without damaging healthy vasculature. CAR T-cells already have shown remarkable clinical success in liquid malignancies like B-cell acute leukemia with response rates above 80%, however, this outstanding efficacy has not yet been translated into the solid tumor setting. The therapy failure can, at least in part, be attributed to the impaired accessibility of the tumor mass due to dysfunctional vasculature and immunosuppressive conditions in the TME. Targeting tumor vessels directly with CAR T-cells could therefore be a good strategy to overcome these issues, which at best, can normalize the defective vasculature and improve drug efficacy in combinational therapy settings. In a recent study Xie et al. (2019) generated VHH-based CAR T-cells targeting EIIIB, an alternatively spliced domain of fibronectin, which is strongly expressed during angiogenesis. Injected EIIIB-targeting CAR T-cells could delay tumor growth and improve survival in immunocompetent mouse models harboring aggressive melanoma, whereas colorectal cancer mouse models did not respond to the treatment. Here, the expression levels of EIIIB in the different tissues had impact on the therapy outcome which again highlights the importance of organ specific vascular markers as well as the impact of organ specific angiogenic activity when targeting tumor vessel formation. Other studies investigated the anti-angiogenic efficacy of TEM8-specific CAR T cells in solid cancer mouse models. TEM8 is one of the first discovered TEC markers and represents a promising target in anti-angiogenic therapy strategies (St Croix et al., 2000). In 2018, a study reported that TEM8-specific CAR T-cells could improve survival and significantly decreased vascularization in triple negative breast cancer mouse models and induced tumor regression in mice with lung metastases (Byrd et al., 2018). A more recent study, however, observed contrasting results where TEM8-sepcific CAR T-cells triggered high toxicity and induced inflammation in lung and spleen when injected into healthy mice (Petrovic et al., 2019). It is suggested that the engineered T-cells cross-reacted with other antigens or targeted TEM8 in healthy tissues, although it is normally expressed at a much lower quantity compared with pathological levels. However, both processes resulted in severe toxicity *in vivo* and again emphasize the need for more adequate, highly specific tumor-vessel exclusive markers that can be targeted with either CAR T-cells or other previously discussed inhibiting molecules.

So far, the main obstacles of anti-angiogenic therapy in NSCLC are evading- or intrinsic resistance mechanisms which still remain elusive. We have discussed a wide array of possible therapies and therapy systems that could improve anti-angiogenic efficacy when combined with standard treatment. The principal goal would be to expand the therapeutical effect of angiogenesis-inhibiting drugs on vessel normalization and render the tumor more vulnerable to additional agents such as chemotherapy or immunotherapy. In a recent study, Hosaka et al. could show that dual angiogenesis inhibition could sensitize resistant off-target tumors to therapy. Therefore they created mouse models of breast cancer or fibrosarcoma, both resistant to anti-VEGF and anti-PDGF treatment due to increased tumor associated expression of bFGF, a molecule which modulates the vasculature via pericyte recruitment in a PDGF-dependent process (Hosaka et al., 2020). Neither anti-VEGF nor anti-PDGF monotherapy had a significant anti-tumor effect on bFGF-positive tumors, but the combination of both agents produced a superior benefit, inhibiting cancer growth by suppressing proliferation and triggering apoptosis of tumor cells. Interestingly, even the pan-blocking of FGF-receptors did not yield a comparable benefit. To explain this unexpected effect, angiogenesis has to be considered as an interacting network of various signaling pathways which cannot be disrupted by blocking a single molecule. In this study the off-target anti-VEGF/PDGF therapy generated a synergistical effect in which PDGF inhibition ablated bFGF-dependent perivascular coverage which further sensitized tumor vessels to anti-VEGF inhibition. These findings demonstrate that the disruption of interacting angiogenic pathways by simultaneously targeting multiple angiogenic factors can provoke a highly potent anti-tumor effect which is able to circumvent mechanisms of therapy resistance, and thus should be considered as new approach to improve neovessel inhibition in cancer. While physiological, as well as pathological vascularization is comprised of diverse molecular pathways, several of which may serve as new targets, some in particular, such as VEGF/VEGFR signaling, represent key players of angiogenesis and should remain an irreplaceable anchor of anti-angiogenic therapy approaches in NSCLC.

## Discussion

Angiogenesis is a main therapeutic concept in oncology, especially in NSCLC, where three approved agents are available in combination with chemotherapy or immunotherapy. Nevertheless, the therapeutic efficacy of the current anti-angiogenic therapies is not satisfying and needs a more personalized/individualized approach. Increasing knowledge in angiogenic processes and non-angiogenic processes that contribute to tumor vascularization, provide precise targets for novel therapy strategies and pave the way for developing new anti-angiogenic treatment concepts that target e.g., TEC metabolism, TEC specific factors, tumor vessel normalization and combinational approaches with CAR T-cells. These therapeutic concepts need to be evaluated for synergistic effects as, in our view, modern anti-angiogenesis represents the concept of shaping the TME rather than being a direct anti-tumor therapy itself. However, these therapeutic strategies are very promising in preclinical setting and the translation into a clinical setting is not only warranted but highly desired. Furthermore, a new horizon of targeted and functional TEC characterization was opened by scRNA-Seq studies, which proved that the tumor vasculature is highly heterogenous and differs from the normal adjacent vasculature more than primarily assumed in terms of metabolic activity, immune suppression and heterogeneity for example. In addition, new synergistic effects of TECs in their role of immunomodulation were identified and induction of HEV formation for immune priming is suggested to be a new therapeutic strategy. Next the organ specific context of the vasculature plays an important role and has to be further studied for better therapy allocation.

In conclusion the concept and goal of anti-angiogenesis in NSCLC in the future can be reshaped by abolishing the traditional vessel priming concept and moving toward a side specific molding of the TME, using the tumor vasculature as a tool, like a trojan horse.

## Author Contributions

SD, HH, EN, AP, and DW developed the concept of the review. SD, HH, and EN drafted the review. DW and AP corrected and reviewed the review. All authors contributed to the article and approved the submitted version.

## Conflict of Interest

The authors declare that the research was conducted in the absence of any commercial or financial relationships that could be construed as a potential conflict of interest. The handling editor LT declared a past co-authorship with one of the authors AP.

## References

[B1] Adighibe O. Micklem K. Campo L. Ferguson M. Harris A. Pozos R. (2006). Is nonangiogenesis a novel pathway for cancer progression? A study using 3-dimensional tumour reconstructions. *Br. J. Cancer* 94 1176–1179. 10.1038/sj.bjc.6603039 16622442 PMC2361259

[B2] Ager A. May M. J. (2015). Understanding high endothelial venules: lessons for cancer immunology. *Oncoimmunology* 4:e1008791. 10.1080/2162402X.2015.1008791 26155419 PMC4485764

[B3] Aguayo A. Manshouri T. O’Brien S. Keating M. Beran M. Koller C. (2001). Clinical relevance of Flt1 and Tie1 angiogenesis receptors expression in B-cell chronic lymphocytic leukemia (CLL). *Leukemia Res.* 25 279–285. 10.1016/S0145-2126(00)00139-9 11248324

[B4] Allen E. Jabouille A. Rivera L. B. Lodewijckx I. Missiaen R. Steri V. (2017). Combined antiangiogenic and anti-PD-L1 therapy stimulates tumor immunity through HEV formation. *Sci. Transl. Med.* 9:eaak9679. 10.1126/scitranslmed.aak9679 28404866 PMC5554432

[B5] Alshangiti A. Chandhoke G. Ellis P. M. (2018). Antiangiogenic therapies in non-small-cell lung cancer. *Curr. Oncol.* 25 S45–S58. 10.3747/co.25.3747 29910647 PMC6001774

[B6] Annan D. A. Maishi N. Soga T. Dawood R. Li C. Kikuchi H. (2019). Carbonic anhydrase 2 (CAII) supports tumor blood endothelial cell survival under lactic acidosis in the tumor microenvironment. *Cell Commun. Signal.* 17:169. 10.1186/s12964-019-0478-4 31847904 PMC6918655

[B7] Auf G. Jabouille A. Guérit S. Pineau R. Delugin M. Bouchecareilh M. (2010). Inositol-requiring enzyme 1alpha is a key regulator of angiogenesis and invasion in malignant glioma. *Proc. Natl. Acad. Sci. U.S.A.* 107 15553–15558. 10.1073/pnas.0914072107 20702765 PMC2932600

[B8] Augustin H. G. Koh G. Y. Thurston G. Alitalo K. (2009). Control of vascular morphogenesis and homeostasis through the angiopoietin-Tie system. *Nat. Rev.. Mol. Cell Biol.* 10 165–177. 10.1038/nrm2639 19234476

[B9] Augustine R. Prasad P. Khalaf I. M. N. (2019). Therapeutic angiogenesis: from conventional approaches to recent nanotechnology-based interventions. *Mater. Sci. Eng. C Mater. biol. Appl.* 97 994–1008. 10.1016/j.msec.2019.01.006 30678987

[B10] Babina I. S. Turner N. C. (2017). Advances and challenges in targeting FGFR signalling in cancer. *Nat. Rev. Cancer* 17 318–332. 10.1038/nrc.2017.8 28303906

[B11] Basile J. R. Castilho R. M. Williams V. P. Gutkind J. S. (2006). Semaphorin 4D provides a link between axon guidance processes and tumor-induced angiogenesis. *Proc. Natl. Acad. Sci. U.S.A.* 103 9017–9022. 10.1073/pnas.0508825103 16754882 PMC1482558

[B12] Bergers G. Benjamin L. E. (2003). Tumorigenesis and the angiogenic switch. *Nat. Rev. Cancer* 3 401–410. 10.1038/nrc1093 12778130

[B13] Bergers G. Hanahan D. (2008). Modes of resistance to anti-angiogenic therapy. *Nat. Rev. Cancer* 8 592–603. 10.1038/nrc2442 18650835 PMC2874834

[B14] Bertolini F. Shaked Y. Mancuso P. Kerbel R. S. (2006). The multifaceted circulating endothelial cell in cancer: towards marker and target identification. *Nat. Rev. Cancer* 6 835–845. 10.1038/nrc1971 17036040

[B15] Betz C. Lenard A. Belting H.-G. Affolter M. (2016). Cell behaviors and dynamics during angiogenesis. *Development* 143 2249–2260. 10.1242/dev.135616 27381223

[B16] Bittner M. Meltzer P. Chen Y. Jiang Y. Seftor E. Hendrix M. (2000). Molecular classification of cutaneous malignant melanoma by gene expression profiling. *Nature* 406 536–540. 10.1038/35020115 10952317

[B17] de Bock K. Georgiadou M. Schoors S. Kuchnio A. Wong B. W. Cantelmo A. R. (2013). Role of PFKFB3-driven glycolysis in vessel sprouting. *Cell* 154 651–663. 10.1016/j.cell.2013.06.037 23911327

[B18] Bridgeman V. L. Vermeulen P. B. Foo S. Bilecz A. Daley F. Kostaras E. (2017). Vessel co-option is common in human lung metastases and mediates resistance to anti-angiogenic therapy in preclinical lung metastasis models. *J. Pathol.* 241 362–374. 10.1002/path.4845 27859259 PMC5248628

[B19] Bruning U. Morales-Rodriguez F. Kalucka J. Goveia J. Taverna F. Queiroz K. C. S. (2018). Impairment of Angiogenesis by Fatty Acid Synthase Inhibition Involves mTOR Malonylation. *Cell Metab.* 28 866.e–880.e. 10.1016/j.cmet.2018.07.019 30146486 PMC8057116

[B20] Byrd T. T. Fousek K. Pignata A. Szot C. Samaha H. Seaman S. (2018). TEM8/ANTXR1-Specific CAR T cells as a targeted therapy for triple-negative breast cancer. *Cancer Res.* 78 489–500. 10.1158/0008-5472.CAN-16-1911 29183891 PMC5771806

[B21] Cantelmo A. R. Conradi L.-C. Brajic A. Goveia J. Kalucka J. Pircher A. (2016). Inhibition of the Glycolytic Activator PFKFB3 in Endothelium Induces Tumor Vessel Normalization. Impairs Metastasis, and Improves Chemotherapy. *Cancer Cell* 30 968–985. 10.1016/j.ccell.2016.10.006 27866851 PMC5675554

[B22] Carlini M. J. Dalurzo M. C. L. Lastiri J. M. Smith D. E. Vasallo B. C. Puricelli L. I. (2010). Mast cell phenotypes and microvessels in non-small cell lung cancer and its prognostic significance. *Hum. Pathol.* 41 697–705. 10.1016/j.humpath.2009.04.029 20040391

[B23] Carmeliet P. Jain R. K. (2011). Molecular mechanisms and clinical applications of angiogenesis. *Nature* 473 298–307. 10.1038/nature10144 21593862 PMC4049445

[B24] Caspani E. M. Crossley P. H. Redondo-Garcia C. Martinez S. (2014). Glioblastoma: a pathogenic crosstalk between tumor cells and pericytes. *PLoS One* 9:e101402. 10.1371/journal.pone.0101402 25032689 PMC4102477

[B25] Chauhan V. P. Jain R. K. (2013). Strategies for advancing cancer nanomedicine. *Nat. Mater.* 12 958–962. 10.1038/nmat3792 24150413 PMC4120281

[B26] Chowdhury N. Vhora I. Patel K. Doddapaneni R. Mondal A. Singh M. (2017). Liposomes co-Loaded with 6-Phosphofructo-2-Kinase/Fructose-2, 6-Biphosphatase 3 (PFKFB3) shRNA Plasmid and Docetaxel for the Treatment of non-small Cell Lung Cancer. *Pharm. Res.* 34 2371–2384. 10.1007/s11095-017-2244-x 28875330 PMC5754003

[B27] Coelho A. L. Gomes M. P. Catarino R. J. Rolfo C. Lopes A. M. Medeiros R. M. (2017). Angiogenesis in NSCLC: is vessel co-option the trunk that sustains the branches? *Oncotarget* 8 39795–39804. 10.18632/oncotarget.7794 26950275 PMC5503654

[B28] Colegio O. R. Chu N.-Q. Szabo A. L. Chu T. Rhebergen A. M. Jairam V. (2014). Functional polarization of tumour-associated macrophages by tumour-derived lactic acid. *Nature* 513 559–563. 10.1038/nature13490 25043024 PMC4301845

[B29] Crawford Y. Kasman I. Yu L. Zhong C. Wu X. Modrusan Z. (2009). PDGF-C mediates the angiogenic and tumorigenic properties of fibroblasts associated with tumors refractory to anti-VEGF treatment. *Cancer Cell* 15 21–34. 10.1016/j.ccr.2008.12.004 19111878

[B30] Crinò L. Dansin E. Garrido P. Griesinger F. Laskin J. Pavlakis N. (2010). Safety and efficacy of first-line bevacizumab-based therapy in advanced non-squamous non-small-cell lung cancer (SAiL, MO19390): a phase 4 study. *Lancet Oncol.* 11 733–740. 10.1016/S1470-2045(10)70151-0 20650686

[B31] Crohns M. Saarelainen S. Laine S. Poussa T. Alho H. Kellokumpu-Lehtinen P. (2010). Cytokines in bronchoalveolar lavage fluid and serum of lung cancer patients during radiotherapy - Association of interleukin-8 and VEGF with survival. *Cytokine* 50 30–36. 10.1016/j.cyto.2009.11.017 20044268

[B32] Delgado-Bellido D. Serrano-Saenz S. Fernández-Cortés M. Oliver F. J. (2017). Vasculogenic mimicry signaling revisited: focus on non-vascular VE-cadherin. *Mol. Cancer* 16:65. 10.1186/s12943-017-0631-x 28320399 PMC5359927

[B33] Dey N. De P. Brian L.-J. (2015). Evading anti-angiogenic therapy: resistance to anti-angiogenic therapy in solid tumors. *Am. J. Transl. Res.* 7 1675–1698. 26692917 PMC4656750

[B34] Döme B. Hendrix M. J. C. Paku S. Tóvári J. Tímár J. (2007). Alternative vascularization mechanisms in cancer: pathology and therapeutic implications. *Am. J. Pathol.* 170 1–15. 10.2353/ajpath.2007.060302 17200177 PMC1762709

[B35] Donnem T. Hu J. Ferguson M. Adighibe O. Snell C. Harris A. L. (2013). Vessel co-option in primary human tumors and metastases: an obstacle to effective anti-angiogenic treatment? *Cancer Med.* 2 427–436. 10.1002/cam4.105 24156015 PMC3799277

[B36] Donnem T. Reynolds A. R. Kuczynski E. A. Gatter K. Vermeulen P. B. Kerbel R. S. (2018). Non-angiogenic tumours and their influence on cancer biology. *Nat. Rev.. Cancer* 18 323–336. 10.1038/nrc.2018.14 29520090

[B37] Dowlati A. Gray R. Sandler A. B. Schiller J. H. Johnson D. H. (2008). Cell adhesion molecules, vascular endothelial growth factor, and basic fibroblast growth factor in patients with non-small cell lung cancer treated with chemotherapy with or without bevacizumab–an Eastern Cooperative Oncology Group Study. *Clin. Cancer Res.* 14 1407–1412. 10.1158/1078-0432.CCR-07-1154 18316562

[B38] Escudier B. Eisen T. Stadler W. M. Szczylik C. Oudard S. Siebels M. (2007). Sorafenib in advanced clear-cell renal-cell carcinoma. *New Eng. J. Med.* 356 125–134. 10.1056/NEJMoa060655 17215530

[B39] Fridman W. H. Pagès F. Sautès-Fridman C. Galon J. (2012). The immune contexture in human tumours: impact on clinical outcome. *Nat. Rev.. Cancer* 12 298–306. 10.1038/nrc3245 22419253

[B40] Fujio Y. Walsh K. (1999). Akt mediates cytoprotection of endothelial cells by vascular endothelial growth factor in an anchorage-dependent manner. *J. Biol. Chem.* 274 16349–16354. 10.1074/jbc.274.23.16349 10347193 PMC3624707

[B41] Garon E. B. Ciuleanu T.-E. Arrieta O. Prabhash K. Syrigos K. N. Goksel T. (2014). Ramucirumab plus docetaxel versus placebo plus docetaxel for second-line treatment of stage IV non-small-cell lung cancer after disease progression on platinum-based therapy (REVEL): a multicentre, double-blind, randomised phase 3 trial. *Lancet* 384 665–673. 10.1016/S0140-6736(14)60845-X 24933332

[B42] Gerber H. P. McMurtrey A. Kowalski J. Yan M. Keyt B. A. Dixit V. (1998). Vascular endothelial growth factor regulates endothelial cell survival through the phosphatidylinositol 3’-kinase/Akt signal transduction pathway. Requirement for Flk-1/KDR activation. *J. Biol. Chem.* 273 30336–30343. 10.1074/jbc.273.46.30336 9804796

[B43] Góth M. I. Hubina E. Raptis S. Nagy G. M. Tóth B. E. (2003). Physiological and pathological angiogenesis in the endocrine system. *Microscopy Res. Tech.* 60 98–106. 10.1002/jemt.10248 12500266

[B44] Goveia J. Rohlenova K. Taverna F. Treps L. Conradi L.-C. Pircher A. (2020). An integrated gene expression landscape profiling approach to identify lung tumor endothelial cell heterogeneity and angiogenic candidates. *Cancer Cell* 37 21.e–36.e. 10.1016/j.ccell.2019.12.001 31935371

[B45] Gridelli C. Castro Carpeno J. de, Dingemans A.-M. C. Griesinger F. Grossi F. (2018). Safety and efficacy of bevacizumab plus standard-of-care treatment beyond disease progression in patients with advanced non-small cell lung cancer: the AvaALL randomized clinical trial. *JAMA Oncol.* 4:e183486. 10.1001/jamaoncol.2018.3486 30177994 PMC6440713

[B46] Györffy B. Lanczky A. Eklund A. C. Denkert C. Budczies J. Li Q. (2010). An online survival analysis tool to rapidly assess the effect of 22,277 genes on breast cancer prognosis using microarray data of 1,809 patients. *Breast Cancer Res Treatment* 123 725–731. 10.1007/s10549-009-0674-9 20020197

[B47] Hall R. D. Le T. M. Haggstrom D. E. Gentzler R. D. (2015). Angiogenesis inhibition as a therapeutic strategy in non-small cell lung cancer (NSCLC). *Transl. Lung Cancer Res.* 4 515–523. 10.3978/j.issn.2218-6751.2015.06.09 26629420 PMC4630516

[B48] Han B. Li K. Wang Q. Zhang L. Shi J. Wang Z. (2018a). Effect of anlotinib as a third-line or further treatment on overall survival of patients with advanced non-small cell lung cancer: the alter 0303 phase 3 randomized clinical trial. *JAMA Oncol.* 4 1569–1575. 10.1001/jamaoncol.2018.3039 30098152 PMC6248083

[B49] Han B. Li K. Zhao Y. Li B. Cheng Y. Zhou J. (2018b). Anlotinib as a third-line therapy in patients with refractory advanced non-small-cell lung cancer: a multicentre, randomised phase II trial (ALTER0302). *Br. J. Cancer* 118 654–661. 10.1038/bjc.2017.478 29438373 PMC5846072

[B50] Hanahan D. Folkman J. (1996). Patterns and Emerging Mechanisms of the Angiogenic Switch during Tumorigenesis. *Cell* 86 353–364. 10.1016/s0092-8674(00)80108-7 8756718

[B51] Hanahan D. Weinberg R. A. (2011). Hallmarks of cancer: the next generation. *Cell* 144 646–674. 10.1016/j.cell.2011.02.013 21376230

[B52] Herbst R. S. Arkenau H.-T. Santana-Davila R. Calvo E. Paz-Ares L. Cassier P. A. (2019). Ramucirumab plus pembrolizumab in patients with previously treated advanced non-small-cell lung cancer, gastro-oesophageal cancer, or urothelial carcinomas (JVDF): a multicohort, non-randomised, open-label, phase 1a/b trial. *Lancet Oncol.* 20 1109–1123. 10.1016/S1470-2045(19)30458-9 31301962

[B53] Hida K. Maishi N. Torii C. Hida Y. (2016). Tumor angiogenesis–characteristics of tumor endothelial cells. *Int. J. Clin. Oncol.* 21 206–212. 10.1007/s10147-016-0957-1 26879652

[B54] Hirono T. Jingushi K. Nagata T. Sato M. Minami K. Aoki M. (2019). MicroRNA-130b functions as an oncomiRNA in non-small cell lung cancer by targeting tissue inhibitor of metalloproteinase-2. *Sci. Rep.* 9:6956. 10.1038/s41598-019-43355-8 31061410 PMC6502853

[B55] Holash J. Wiegand S. J. Yancopoulos G. D. (1999). New model of tumor angiogenesis: dynamic balance between vessel regression and growth mediated by angiopoietins and VEGF. *Oncogene* 18 5356–5362. 10.1038/sj.onc.1203035 10498889

[B56] Hosaka K. Yang Y. Seki T. Du Q. Jing X. He X. (2020). Therapeutic paradigm of dual targeting VEGF and PDGF for effectively treating FGF-2 off-target tumors. *Nat. Commun.* 11:3704. 10.1038/s41467-020-17525-6 32709869 PMC7382445

[B57] Hsu Y.-L. Hung J.-Y. Chang W.-A. Lin Y.-S. Pan Y.-C. Tsai P.-H. (2017). Hypoxic lung cancer-secreted exosomal miR-23a increased angiogenesis and vascular permeability by targeting prolyl hydroxylase and tight junction protein ZO-1. *Oncogene* 36 4929–4942. 10.1038/onc.2017.105 28436951

[B58] Hu T. Shen H. Huang H. Yang Z. Zhou Y. Zhao G. (2020). Cholesterol-lowering drug pitavastatin targets lung cancer and angiogenesis via suppressing prenylation-dependent Ras/Raf/MEK and PI3K/Akt/mTOR signaling. *Anti Cancer Drugs* 31 377–384. 10.1097/CAD.0000000000000885 32011362

[B59] Huang B. Lei Z. Zhang G.-M. Li D. Song C. Li B. (2008). SCF-mediated mast cell infiltration and activation exacerbate the inflammation and immunosuppression in tumor microenvironment. *Blood* 112 1269–1279. 10.1182/blood-2008-03-147033 18524989 PMC2515142

[B60] Ibaraki T. Muramatsu M. Takai S. Jin D. Maruyama H. Orino T. (2005). The relationship of tryptase- and chymase-positive mast cells to angiogenesis in stage I non-small cell lung cancer. *Eur. J. Cardio-Thorac. Surg.* 28 617–621. 10.1016/j.ejcts.2005.06.020 16125954

[B61] Janning M. Loges S. (2018). Anti-Angiogenics: their Value in Lung Cancer Therapy. *Oncol. Res. Treat.* 41 172–180. 10.1159/000488119 29631257

[B62] Jayson G. C. Kerbel R. Ellis L. M. Harris A. L. (2016). Antiangiogenic therapy in oncology: current status and future directions. *Lancet* 388 518–529. 10.1016/S0140-6736(15)01088-0 26853587

[B63] Jensen L. D. (2016). When tumors are (co-)opting to resist anti-angiogenic treatment. *Transl. Cancer Res.* 5 S1433–S1436. 10.21037/tcr.2016.12.35

[B64] Johnson D. H. Fehrenbacher L. Novotny W. F. Herbst R. S. Nemunaitis J. J. Jablons D. M. (2004). Randomized phase II trial comparing bevacizumab plus carboplatin and paclitaxel with carboplatin and paclitaxel alone in previously untreated locally advanced or metastatic non-small-cell lung cancer. *J. Clin Oncol* 22 2184–2191. 10.1200/JCO.2004.11.022 15169807

[B65] Kaipainen A. Vlaykova T. Hatva E. Böhling T. Jekunen A. Pyrhönen S. (1994). Enhanced expression of the tie receptor tyrosine kinase mesenger RNA in the vascular endothelium of metastatic melanomas. *Cancer Res.* 54 6571–6577. 7987857

[B66] Kessenbrock K. Plaks V. Werb Z. (2010). Matrix metalloproteinases: regulators of the tumor microenvironment. *Cell* 141 52–67. 10.1016/j.cell.2010.03.015 20371345 PMC2862057

[B67] Kim M. Allen B. Korhonen E. A. Nitschké M. Yang H. W. Baluk P. (2016). Opposing actions of angiopoietin-2 on Tie2 signaling and FOXO1 activation. *J. Clin. Invest.* 126 3511–3525. 10.1172/JCI84871 27548529 PMC5004955

[B68] Korhonen E. A. Lampinen A. Giri H. Anisimov A. Kim M. Allen B. (2016). Tie1 controls angiopoietin function in vascular remodeling and inflammation. *J. Clin. Invest.* 126 3495–3510. 10.1172/JCI84923 27548530 PMC5004934

[B69] Kut C. Mac Gabhann F. Popel A. S. (2007). Where is VEGF in the body? A meta-analysis of VEGF distribution in cancer. *Br. J. Cancer* 97 978–985. 10.1038/sj.bjc.6603923 17912242 PMC2360423

[B70] La Porta S. Roth L. Singhal M. Mogler C. Spegg C. Schieb B. (2018). Endothelial Tie1-mediated angiogenesis and vascular abnormalization promote tumor progression and metastasis. *J. Clin. Invest.* 128 834–845. 10.1172/JCI94674 29355844 PMC5785248

[B71] Lambrechts D. Wauters E. Boeckx B. Aibar S. Nittner D. Burton O. (2018). Phenotype molding of stromal cells in the lung tumor microenvironment. *Nat. Med.* 24 1277–1289. 10.1038/s41591-018-0096-5 29988129

[B72] Lee J. G. Wu R. (2015). Erlotinib-cisplatin combination inhibits growth and angiogenesis through c-MYC and HIF-1α in EGFR-mutated lung cancer in vitro and in vivo. *Neoplasia* 17 190–200. 10.1016/j.neo.2014.12.008 25748238 PMC4351293

[B73] Li D. Xie K. Zhang L. Yao X. Li H. Xu Q. (2016). Dual blockade of vascular endothelial growth factor (VEGF) and basic fibroblast growth factor (FGF-2) exhibits potent anti-angiogenic effects. *Cancer Lett.* 377 164–173. 10.1016/j.canlet.2016.04.036 27130666

[B74] Li X. Sun X. Carmeliet P. (2019). Hallmarks of endothelial cell metabolism in health and disease. *Cell Metab.* 30 414–433. 10.1016/j.cmet.2019.08.011 31484054

[B75] Liu H. Chen Y. Li Y. Li C. Qin T. Bai M. (2019). miR-195 suppresses metastasis and angiogenesis of squamous cell lung cancer by inhibiting the expression of VEGF. *Mol. Med. Rep.* 20 2625–2632. 10.3892/mmr.2019.10496 31322197 PMC6691228

[B76] Liu T. Ma W. Xu H. Huang M. Zhang D. He Z. (2018). PDGF-mediated mesenchymal transformation renders endothelial resistance to anti-VEGF treatment in glioblastoma. *Nat. Commun.* 9:3439. 10.1038/s41467-018-05982-z 30150753 PMC6110798

[B77] Lu J. Xu W. Qian J. Wang S. Zhang B. Zhang L. (2019a). Transcriptome profiling analysis reveals that CXCL2 is involved in anlotinib resistance in human lung cancer cells. *BMC Med. Genomics* 12:38. 10.1186/s12920-019-0482-y 30871526 PMC6416828

[B78] Lu J. Zhong H. Chu T. Zhang X. Li R. Sun J. (2019b). Role of anlotinib-induced CCL2 decrease in anti-angiogenesis and response prediction for nonsmall cell lung cancer therapy. *Eur. Respirat. J.* 53:1801562. 10.1183/13993003.01562-2018 30578392

[B79] Lyu J. Yang E. J. Head S. A. Ai N. Zhang B. Wu C. (2017). Pharmacological blockade of cholesterol trafficking by cepharanthine in endothelial cells suppresses angiogenesis and tumor growth. *Cancer Lett.* 409 91–103. 10.1016/j.canlet.2017.09.009 28923401 PMC5634947

[B80] Maniotis A. J. Folberg R. Hess A. Seftor E. A. Gardner L. M. G. Pe’er J. (1999). Vascular channel formation by human melanoma cells in Vivo and in Vitro: vasculogenic Mimicry. *Am. J. Pathol.* 155 739–752. 10.1016/S0002-9440(10)65173-5 10487832 PMC1866899

[B81] Martinet L. Girard J.-P. (2013). Regulation of tumor-associated high-endothelial venules by dendritic cells: a new opportunity to promote lymphocyte infiltration into breast cancer? *Oncoimmunology* 2:e26470. 10.4161/onci.26470 24482745 PMC3897501

[B82] Mok T. Gorbunova V. Juhasz E. Szima B. Burdaeva O. Orlov S. (2014). A correlative biomarker analysis of the combination of bevacizumab and carboplatin-based chemotherapy for advanced nonsquamous non-small-cell lung cancer: results of the phase II randomized ABIGAIL study (BO21015). *J. Thoracic Oncol.* 9 848–855. 10.1097/JTO.0000000000000160 24807156

[B83] Montana V. Sontheimer H. (2011). Bradykinin promotes the chemotactic invasion of primary brain tumors. *J. Neurosci.* 31 4858–4867. 10.1523/JNEUROSCI.3825-10.2011 21451024 PMC3096850

[B84] Nakagawa K. Garon E. B. Seto T. Nishio M. Ponce Aix S. Paz-Ares L. (2019). Ramucirumab plus erlotinib in patients with untreated, EGFR-mutated, advanced non-small-cell lung cancer (RELAY): a randomised, double-blind, placebo-controlled, phase 3 trial. *Lancet Oncol.* 20 1655–1669. 10.1016/S1470-2045(19)30634-5 31591063

[B85] Novello S. Kaiser R. Mellemgaard A. Douillard J.-Y. Orlov S. Krzakowski M. (2015). Analysis of patient-reported outcomes from the LUME-Lung 1 trial: a randomised, double-blind, placebo-controlled, Phase III study of second-line nintedanib in patients with advanced non-small cell lung cancer. *Eur. J. Cancer* 51 317–326. 10.1016/j.ejca.2014.11.015 25534294

[B86] Olson O. C. Joyce J. A. (2015). Cysteine cathepsin proteases: regulators of cancer progression and therapeutic response. *Nat. Rev. Cancer* 15 712–729. 10.1038/nrc4027 26597527

[B87] Overed-Sayer C. Miranda E. Dunmore R. Liarte Marin E. Beloki L. Rassl D. (2020). Inhibition of mast cells: a novel mechanism by which nintedanib may elicit anti-fibrotic effects. *Thorax* 75 754–763. 10.1136/thoraxjnl-2019-214000 32709610 PMC7476277

[B88] Paulis Y. W. J. Soetekouw P. M. M. B. Verheul H. M. W. Tjan-Heijnen V. C. G. Griffioen A. W. (2010). Signalling pathways in vasculogenic mimicry. *Biochim. Biophys. Acta* 1806 18–28. 10.1016/j.bbcan.2010.01.001 20079807

[B89] Peterson T. E. Kirkpatrick N. D. Huang Y. Farrar C. T. Marijt K. A. Kloepper J. (2016). Dual inhibition of Ang-2 and VEGF receptors normalizes tumor vasculature and prolongs survival in glioblastoma by altering macrophages. *Proc. Natl. Acad. Sci. U.S.A.* 113 4470–4475. 10.1073/pnas.1525349113 27044097 PMC4843449

[B90] Petrovic K. Robinson J. Whitworth K. Jinks E. Shaaban A. Lee S. P. (2019). TEM8/ANTXR1-specific CAR T cells mediate toxicity in vivo. *PLoS One* 14:e0224015. 10.1371/journal.pone.0224015 31622431 PMC6797195

[B91] Pezzella F. Pastorino U. Tagliabue E. Andreola S. Sozzi G. Gasparini G. (1997). Non-small-cell lung carcinoma tumor growth without morphological evidence of neo-angiogenesis. *Am. J. Pathol.* 151 1417–1423. 9358768 PMC1858069

[B92] Phung T. L. Ziv K. Dabydeen D. Eyiah-Mensah G. Riveros M. Perruzzi C. (2006). Pathological angiogenesis is induced by sustained Akt signaling and inhibited by rapamycin. *Cancer Cell* 10 159–170. 10.1016/j.ccr.2006.07.003 16904613 PMC2531257

[B93] Pinto M. P. Sotomayor P. Carrasco-Avino G. Corvalan A. H. Owen G. I. (2016). Escaping antiangiogenic therapy: strategies employed by cancer cells. *Int. J. Mol. Sci.* 17:1489. 10.3390/ijms17091489 27608016 PMC5037767

[B94] Priceman S. J. Sung J. L. Shaposhnik Z. Burton J. B. Torres-Collado A. X. Moughon D. L. (2010). Targeting distinct tumor-infiltrating myeloid cells by inhibiting CSF-1 receptor: combating tumor evasion of antiangiogenic therapy. *Blood* 115 1461–1471. 10.1182/blood-2009-08-237412 20008303 PMC2826767

[B95] Qiu L. Zhao X. Shi W. Sun S. Zhang G. Sun Q. (2020). Real-world treatment efficacy of anti-programmed death-1 combined with anti-angiogenesis therapy in non-small cell lung cancer patients. *Medicine* 99:e20545. 10.1097/MD.0000000000020545 32541476 PMC7302578

[B96] Rafii S. Butler J. M. Ding B.-S. (2016). Angiocrine functions of organ-specific endothelial cells. *Nature* 529 316–325. 10.1038/nature17040 26791722 PMC4878406

[B97] Rafii S. Lyden D. Benezra R. Hattori K. Heissig B. (2002). Vascular and haematopoietic stem cells: novel targets for anti-angiogenesis therapy? *Nat. Rev. Cancer* 2 826–835. 10.1038/nrc925 12415253

[B98] Raica M. Cimpean A. M. Ribatti D. (2009). Angiogenesis in pre-malignant conditions. *Eur. J. Cancer* 45 1924–1934. 10.1016/j.ejca.2009.04.007 19406633

[B99] Reck M. Kaiser R. Mellemgaard A. Douillard J.-Y. Orlov S. Krzakowski M. (2014). Docetaxel plus nintedanib versus docetaxel plus placebo in patients with previously treated non-small-cell lung cancer (LUME-Lung 1): a phase 3, double-blind, randomised controlled trial. *Lancet Oncol.* 15 143–155. 10.1016/S1470-2045(13)70586-2 24411639

[B100] Reck M. Pawel J. von, Zatloukal P. Ramlau R. Gorbounova V. (2009). Phase III trial of cisplatin plus gemcitabine with either placebo or bevacizumab as first-line therapy for nonsquamous non-small-cell lung cancer: AVAil. *J. Clin. Oncol.* 27 1227–1234. 10.1200/JCO.2007.14.5466 19188680

[B101] Reck M. Pawel J. von, Zatloukal P. Ramlau R. Gorbounova V. (2010). Overall survival with cisplatin-gemcitabine and bevacizumab or placebo as first-line therapy for nonsquamous non-small-cell lung cancer: results from a randomised phase III trial (AVAiL). *Ann. Oncol.* 21 1804–1809. 10.1093/annonc/mdq020 20150572 PMC2924992

[B102] Reck M. Paz-Ares L. Bidoli P. Cappuzzo F. Dakhil S. Moro-Sibilot D. (2017). Outcomes in patients with aggressive or refractory disease from REVEL: a randomized phase III study of docetaxel with ramucirumab or placebo for second-line treatment of stage IV non-small-cell lung cancer. *Lung Cancer* 112 181–187. 10.1016/j.lungcan.2017.07.038 29191593

[B103] Ribatti D. Crivellato E. (2012). Mast cells, angiogenesis, and tumour growth. *Biochim. Biophys. Acta* 1822 2–8. 10.1016/j.bbadis.2010.11.010 21130163

[B104] Ribatti D. Ranieri G. (2015). Tryptase, a novel angiogenic factor stored in mast cell granules. *Exp. Cell Res.* 332 157–162. 10.1016/j.yexcr.2014.11.014 25478999

[B105] Rigamonti N. Kadioglu E. Keklikoglou I. Wyser Rmili C. Leow C. C. de Palma M. (2014). Role of angiopoietin-2 in adaptive tumor resistance to VEGF signaling blockade. *Cell Rep.* 8 696–706. 10.1016/j.celrep.2014.06.059 25088418

[B106] Rodríguez Garzotto A. Díaz-García C. V. Agudo-López A. Prieto García E. Ponce S. López-Martín J. A. (2016). Blood-based biomarkers for monitoring antiangiogenic therapy in non-small cell lung cancer. *Med. Oncol.* 33:105. 10.1007/s12032-016-0824-y 27568331

[B107] Rohlenova K. Goveia J. García-Caballero M. Subramanian A. Kalucka J. Treps L. (2020). Single-Cell RNA Sequencing Maps Endothelial Metabolic Plasticity in Pathological Angiogenesis. *Cell Metab.* 31 862.e14–877.e14. 10.1016/j.cmet.2020.03.009 32268117

[B108] Saito H. Fukuhara T. Furuya N. Watanabe K. Sugawara S. Iwasawa S. (2019). Erlotinib plus bevacizumab versus erlotinib alone in patients with EGFR-positive advanced non-squamous non-small-cell lung cancer (NEJ026): interim analysis of an open-label, randomised, multicentre, phase 3 trial. *Lancet Oncol.* 20 625–635. 10.1016/S1470-2045(19)30035-X 30975627

[B109] Sakurai Y. Akita H. Harashima H. (2019). Targeting Tumor Endothelial Cells with Nanoparticles. *Int. J. Mol. Sci.* 20:5819. 10.3390/ijms20235819 31756900 PMC6928777

[B110] Sanctis F. de, Ugel S. Facciponte J. Facciabene A. (2018). The dark side of tumor-associated endothelial cells. *Semin. Immunol.* 35 35–47. 10.1016/j.smim.2018.02.002 29490888

[B111] Sandler A. Gray R. Perry M. C. Brahmer J. Schiller J. H. Dowlati A. (2006). Paclitaxel-carboplatin alone or with bevacizumab for non-small-cell lung cancer. *New Engl. J. Med.* 355 2542–2550. 10.1056/NEJMoa061884 17167137

[B112] Sato T. N. Tozawa Y. Deutsch U. Wolburg-Buchholz K. Fujiwara Y. Gendron-Maguire M. (1995). Distinct roles of the receptor tyrosine kinases Tie-1 and Tie-2 in blood vessel formation. *Nature* 376 70–74. 10.1038/376070a0 7596437

[B113] Schmittnaegel M. Rigamonti N. Kadioglu E. Cassará A. Wyser Rmili C. Kiialainen A. (2017). Dual angiopoietin-2 and VEGFA inhibition elicits antitumor immunity that is enhanced by PD-1 checkpoint blockade. *Sci. Transl. Med.* 9:eaak9670. 10.1126/scitranslmed.aak9670 28404865

[B114] Schwaederlé M. Lazar V. Validire P. Hansson J. Lacroix L. Soria J.-C. (2015). VEGF-A expression correlates with TP53 mutations in non-small cell lung cancer: implications for antiangiogenesis therapy. *Cancer Res.* 75 1187–1190. 10.1158/0008-5472.CAN-14-2305 25672981

[B115] Seftor E. A. Meltzer P. S. Kirschmann D. A. Pe’er J. Maniotis A. J. Trent J. M. (2002a). Molecular determinants of human uveal melanoma invasion and metastasis. *Clin. Exp. Metastasis* 19 233–246. 10.1023/a:1015591624171 12067204

[B116] Seftor E. A. Meltzer P. S. Schatteman G. C. Gruman L. M. Hess A. R. Kirschmann D. A. (2002b). Expression of multiple molecular phenotypes by aggressive melanoma tumor cells: role in vasculogenic mimicry. *Crit. Rev. Oncol.* 44 17–27. 10.1016/S1040-8428(01)00199-8 12398997

[B117] Seftor R. E. Seftor E. A. Koshikawa N. Meltzer P. S. Gardner L. M. Bilban M. (2001). Cooperative interactions of laminin 5 gamma2 chain, matrix metalloproteinase-2, and membrane type-1-matrix/metalloproteinase are required for mimicry of embryonic vasculogenesis by aggressive melanoma. *Cancer Res.* 61 6322–6327. 11522618

[B118] Senger D. R. Davis G. E. (2011). Angiogenesis. *Cold Spring Harbor Perspect. Biol.* 3:a005090. 10.1101/cshperspect.a005090 21807843 PMC3140681

[B119] Seshadri D. R. Ramamurthi A. (2018). Nanotherapeutics to modulate the compromised micro-environment for lung cancers and chronic obstructive pulmonary disease. *Front. Pharmacol.* 9:759. 10.3389/fphar.2018.00759 30061830 PMC6054931

[B120] Seto T. Kato T. Nishio M. Goto K. Atagi S. Hosomi Y. (2014). Erlotinib alone or with bevacizumab as first-line therapy in patients with advanced non-squamous non-small-cell lung cancer harbouring EGFR mutations (JO25567): an open-label, randomised, multicentre, phase 2 study. *Lancet Oncol.* 15 1236–1244. 10.1016/S1470-2045(14)70381-X 25175099

[B121] Shahid I. AlMalki W. H. AlRabia M. W. Ahmed M. Imam M. T. Saifullah M. K. (2017). “Recent Advances in Angiogenesis Assessment Methods and their Clinical Applications,” in *Physiologic and Pathologic Angiogenesis. Signaling Mechanisms and Targeted Therapy*, eds Simionescu D. Simionescu A. (Rijeka: InTech).

[B122] Shi Y. Du L. Lin L. Wang Y. (2017). Tumour-associated mesenchymal stem/stromal cells: emerging therapeutic targets. *Nature reviews*. *Drug Discov.* 16 35–52. 10.1038/nrd.2016.193 27811929

[B123] Shibata Y. Kobayashi N. Sato T. Nakashima K. Kaneko T. (2020). The clinical significance of CXCL16 in the treatment of advanced non-small cell lung cancer. *Thorac. Cancer* 11 1258–1264. 10.1111/1759-7714.13387 32163231 PMC7180569

[B124] Shojaei F. Wu X. Malik A. K. Zhong C. Baldwin M. E. Schanz S. (2007). Tumor refractoriness to anti-VEGF treatment is mediated by CD11b+Gr1+ myeloid cells. *Nat. Biotechnol.* 25 911–920. 10.1038/nbt1323 17664940

[B125] Socinski M. A. Jotte R. M. Cappuzzo F. Orlandi F. Stroyakovskiy D. Nogami N. (2018). Atezolizumab for First-Line Treatment of Metastatic Nonsquamous NSCLC. *New Engl. J. Med.* 378 2288–2301. 10.1056/NEJMoa1716948 29863955

[B126] Soria J.-C. Mauguen A. Reck M. Sandler A. B. Saijo N. Johnson D. H. (2013). Systematic review and meta-analysis of randomised, phase II/III trials adding bevacizumab to platinum-based chemotherapy as first-line treatment in patients with advanced non-small-cell lung cancer. *Ann. Oncol.* 24 20–30. 10.1093/annonc/mds590 23180113

[B127] Soucek L. Lawlor E. R. Soto D. Shchors K. Swigart L. B. Evan G. I. (2007). Mast cells are required for angiogenesis and macroscopic expansion of Myc-induced pancreatic islet tumors. *Nat. Med.* 13 1211–1218. 10.1038/nm1649 17906636

[B128] St Croix B. Rago C. Velculescu V. Traverso G. Romans K. E. Montgomery E. (2000). Genes expressed in human tumor endothelium. *Science* 289 1197–1202. 10.1126/science.289.5482.1197 10947988

[B129] Stryker Z. I. Rajabi M. Davis P. J. Mousa S. A. (2019). Evaluation of angiogenesis assays. *Biomedicines* 7:37. 10.3390/biomedicines7020037 31100863 PMC6631830

[B130] Sun H. Zhang D. Yao Z. Lin X. Liu J. Gu Q. (2017). Anti-angiogenic treatment promotes triple-negative breast cancer invasion via vasculogenic mimicry. *Cancer Biol. Ther.* 18 205–213. 10.1080/15384047.2017.1294288 28278077 PMC5450737

[B131] Sun L.-L. Li W.-D. Lei F.-R. Li X.-Q. (2018). The regulatory role of microRNAs in angiogenesis-related diseases. *J. Cell. Mol. Med.* 22 4568–4587. 10.1111/jcmm.13700 29956461 PMC6156236

[B132] Takeuchi A. Oguri T. Yamashita Y. Sone K. Fukuda S. Takakuwa O. (2018). TTF-1 expression predicts the merit of additional antiangiogenic treatment in non-squamous non-small cell lung cancer. *Anticancer Res.* 38 5489–5495. 10.21873/anticanres.12882 30194207

[B133] Teleanu R. I. Chircov C. Grumezescu A. M. Teleanu D. M. (2019). Tumor angiogenesis and anti-angiogenic strategies for cancer treatment. *J. Clin. Med.* 9:84. 10.3390/jcm9010084 31905724 PMC7020037

[B134] Uribesalgo I. Hoffmann D. Zhang Y. Kavirayani A. Lazovic J. Berta J. (2019). Apelin inhibition prevents resistance and metastasis associated with anti-angiogenic therapy. *EMBO Mol. Med.* 11:e9266. 10.15252/emmm.201809266 31267692 PMC6685079

[B135] van der Veldt A. A. M. Lubberink M. Bahce I. Walraven M. Boer M. P. de (2012). Rapid decrease in delivery of chemotherapy to tumors after anti-VEGF therapy: implications for scheduling of anti-angiogenic drugs. *Cancer Cell* 21 82–91. 10.1016/j.ccr.2011.11.023 22264790

[B136] Webb N. J. Bottomley M. J. Watson C. J. Brenchley P. E. (1998). Vascular endothelial growth factor (VEGF) is released from platelets during blood clotting: implications for measurement of circulating VEGF levels in clinical disease. *Clin. Sci*. 94 395–404. 10.1042/cs0940395 9640345

[B137] Williamson S. C. Metcalf R. L. Trapani F. Mohan S. Antonello J. Abbott B. (2016). Vasculogenic mimicry in small cell lung cancer. *Nat. Commun.* 7:13322. 10.1038/ncomms13322 27827359 PMC5105195

[B138] Wroblewski M. Bauer R. Cubas Córdova M. Udonta F. Ben-Batalla I. Legler K. (2017). Mast cells decrease efficacy of anti-angiogenic therapy by secreting matrix-degrading granzyme B. *Nat. Commun.* 8:269. 10.1038/s41467-017-00327-8 28814715 PMC5559596

[B139] Xia Y. Cai X.-Y. Fan J.-Q. Zhang L.-L. Ren J.-H. Li Z.-Y. (2019). The role of sema4D in vasculogenic mimicry formation in non-small cell lung cancer and the underlying mechanisms. *Int. J. Cancer* 144 2227–2238. 10.1002/ijc.31958 30374974

[B140] Xie Y. J. Dougan M. Jailkhani N. Ingram J. Fang T. Kummer L. (2019). Nanobody-based CAR T cells that target the tumor microenvironment inhibit the growth of solid tumors in immunocompetent mice. *Proc. Natl. Acad. Sci. U.S.A.* 116 7624–7631. 10.1073/pnas.1817147116 30936321 PMC6475367

[B141] Yamamoto N. Seto T. Nishio M. Goto K. Okamoto I. Yamanaka T. (2018). Erlotinib plus bevacizumab (EB) versus erlotinib alone (E) as first-line treatment for advanced EGFR mutation–positive non-squamous non–small-cell lung cancer (NSCLC): Survival follow-up results of JO25567. *J. Clin. Oncol.* 36:9007 10.1200/JCO.2018.36.15_suppl.9007 33279874

[B142] Yang Y. Zhang Y. Iwamoto H. Hosaka K. Seki T. Andersson P. (2016). Discontinuation of anti-VEGF cancer therapy promotes metastasis through a liver revascularization mechanism. *Nat. Commun.* 7:12680. 10.1038/ncomms12680 27580750 PMC5025794

[B143] Yu D.-H. Lu Q. Xie J. Fang C. Chen H.-Z. (2010). Peptide-conjugated biodegradable nanoparticles as a carrier to target paclitaxel to tumor neovasculature. *Biomaterials* 31 2278–2292. 10.1016/j.biomaterials.2009.11.047 20053444

[B144] Zaman K. Driscoll R. Hahn D. Werffeli P. Goodman S. L. Bauer J. (2006). Monitoring multiple angiogenesis-related molecules in the blood of cancer patients shows a correlation between VEGF-A and MMP-9 levels before treatment and divergent changes after surgical vs. *conservative therapy*. *Int. J. Cancer* 118 755–764. 10.1002/ijc.21408 16114015

[B145] Zhang L. Deng Y. Zhang Y. Liu C. Zhang S. Zhu W. (2020). The Design, Characterizations, and Tumor Angiogenesis Inhibition of a Multi-Epitope Peptibody With bFGF/VEGFA. *Front. Oncol.* 10:1190. 10.3389/fonc.2020.01190 32766160 PMC7379876

